# Supplementary material for: Testosterone replacement in young male cancer survivors: A 6-month double-blind randomised placebo-controlled trial
Source: PLoS Med. 2019 Nov 12;16(11):e1002960. doi: 10.1371/journal.pmed.1002960 (PMC6850530; doi:10.1371/journal.pmed.1002960)
Supplement: S1 Text — (DOCX) [file pmed.1002960.s001.docx]

**Testosterone Replacement in Young Male cancer Survivors**

ISRCTN: 70274195

EudraCT Number: 2011-000677-31

Version 6.8, 4th July 2014

**Chief Investigator** Professor Richard Ross Professor of Endocrinology Room M112

Royal Hallamshire Hospital Glossop Road

Sheffield S10 2JF

Tel: 0114 2712052

Ema[il: r.j.ross@sheffield.ac.uk](mailto:r.j.ross@sheffield.ac.uk)

**Sponsor** Sheffield Teaching Hospitals NHS Foundation Trust Research Department

1st Floor

11 Broomfield Road Sheffield

S10 2SE

Tel: 0114 226 5938

*The Sponsor and CTRU accept no responsibility for the accuracy of additional documentation or instructions developed by collaborating or third party organisations, from the content of this protocol.*

# KEY CONTACTS

##### Please direct any trial specific questions to the Trial Manager / Co-ordinator in the first instance.

**Chief Investigator** Professor Richard Ross Head of Department Endocrinology

Room M112

Royal Hallamshire Hospital Glossop Road

Sheffield S10 2JF

Email: [r.j.ross@sheffield.ac.uk](mailto:r.j.ross@sheffield.ac.uk) Telephone: 0114 2711634

#### Co-Investigators (for clinical queries in the absence of the Chief Investigator)

Dr Jennifer Walsh Consultant Physician

Endocrinology and Metabolic Bone Medicine Metabolic Bone Centre

Sorby, Northern General Hospital Herries Road

Sheffield S5 7AU

Email: [jennifer.walsh2@sth.nhs.uk](mailto:jennifer.walsh2@sth.nhs.uk) Telephone: 0114 2266941

Dr Diana Greenfield Nurse Consultant Weston Park Hospital Whitham Road Sheffield

S10 2SJ

Email: [diana.greenfield@sth.nhs.uk](mailto:diana.greenfield@sth.nhs.uk) Telephone: 0114 2265592

Dr John Snowden Consultant Haematologist Royal Hallamshire Hospital Glossop Road

Sheffield S10 2JF

Email: [john.snowden@sth.nhs.uk](mailto:john.snowden@sth.nhs.uk) Telephone: 0114 2713411

Professor Rob Coleman Head of Unit

Medical Oncology Weston Park Hospital Whitham Road Sheffield

S10 2SJ

Email: [r.e.coleman@sheffield.ac.uk](mailto:r.e.coleman@sheffield.ac.uk) Telephone: 0114 2265213

Dr Robert Huddart

Reader in Urological Oncology Institute of Cancer Research 15 Cotswold Rd

Belmont Sutton Surrey SM2 5NG

Email: [robert.huddart@icr.ac.uk](mailto:robert.huddart@icr.ac.uk) Telephone: 020 8661 3529

#### CTRU Staff

Ms Alexandra Smith

Head of Trial Management - Solid Tumours Leeds Institute of Clinical Trials Research Clinical Trials Research Unit

University of Leeds Leeds

LS2 9JT

Tel: 0113 343 2657

Fax: 0113 343 1471

Email: [a.f.smith@leeds.ac.uk](mailto:a.f.smith@leeds.ac.uk)

Mrs Jayne Swain

Trial Management Contact (for site set-up, regulatory and governance queries) Leeds Institute of Clinical Trials Research

Clinical Trials Research Unit University of Leeds

Leeds LS2 9JT

Tel.: 0113 343 8091

Fax: 0113 343 4345

Email: [ctru_tryms@leeds.ac.uk](mailto:ctru_tryms@leeds.ac.uk)

Mrs Emma Best

Data Management Contact (for eligibility and data related queries) Leeds Institute of Clinical Trials Research

Clinical Trials Research Unit University of Leeds

Leeds LS2 9JT

Tel: 0113 343 3914

Fax: 0113 343 6774

Email: [ctru_tryms@leeds.ac.uk](mailto:ctru_tryms@leeds.ac.uk)

Miss Isabelle Smith Medical Statistician

Leeds Institute of Clinical Trials Research Clinical Trials Research Unit

University of Leeds Leeds

LS2 9JT

Tel: 0113 343 1481

Fax: 0113 343 1471

Email: [i.l.Smith@leeds.ac.uk](mailto:i.l.Smith@leeds.ac.uk)

Mrs Helen Marshall Principal Statistician

Leeds Institute of Clinical Trials Research Clinical Trials Research Unit

University of Leeds Leeds

LS2 9JT

Telephone: 0113 343 1481

Fax: 0113 343 1471

Email: [h.c.marshall@leeds.ac.uk](mailto:h.c.marshall@leeds.ac.uk)

Miss Sarah Brown Principal Statistician

Leeds Institute of Clinical Trials Research Clinical Trials Research Unit

University of Leeds Leeds

LS2 9JT

Tel: 0113 343 1475

Fax: 0113 343 1471

Ema[il: medsbro@leeds.ac.uk](mailto:medsbro@leeds.ac.uk)

Professor Julia Brown

Director of Clinical Trials Research Unit Professor of Clinical Trials Research Leeds Institute of Clinical Trials Research Clinical Trials Research Unit

University of Leeds Leeds

LS2 9JT

Tel: 0113 343 1477

Fax: 0113 343 1471

Email: [j.m.b.brown@leeds.ac.uk](mailto:j.m.b.brown@leeds.ac.uk)

#### Laboratory Contact

Lynn Male

Department of Laboratory Medicine Northern General Hospital

Herries Road Sheffield

S5 7AU

Tel: 2669471

Ema[il: lynn.male@sth.nhs.uk](mailto:lynn.male@sth.nhs.uk)

#### Trial Registration, Randomisation and Replenishing Stock

##### Only authorised members of staff from hospital sites with appropriate trial approvals have permission to register and randomise participants into the trial and to request Kit Codes for dispensing at week 13 or replacement of stock. Please note that these systems are automated and you will need an Authorisation Code and Personal Identification Number (PIN) to access them. Please see sections 8.4, 8.5, 9.1.2 and 9.8 for further information.

#### Participant Registration

Tel: 0113 343 8265

#### Randomisation

Tel.: 0113 343 8265

#### Week 13 Dispensing and Replacing Stock

Tel: 0113 343 8265

#### Reporting Serious Adverse Events (SAEs) and Suspected Serious Adverse Reactions (SSARs)

Complete the SAE Report Case Report Form (CRF) for all SAEs and SSARs occurring in the trial and fax to the CTRU within 24 hours of becoming aware of the event:

Fax: CTRU: 0113 343 6774

Further information can be found in section 12. Any issues should be discussed with the Data Manager at CTRU.

A receipt will be sent for all received SAEs and SSARs. ***If a receipt is not received within 2 working days contact the Data Manager / Senior Trial Co-ordinator at CTRU.***

#### Reporting Suspected Unexpected Serious Adverse Reactions (SUSARs)

Complete the SUSAR Report CRF for all SUSARs occurring in the trial and fax to the CTRU within 24 hours of becoming aware of the event:

Fax: CTRU: 0113 343 6774

Further information can be found in section 12. Any issues should be discussed with the Data Manager.

A receipt will be sent for all received SUSARs. ***If a receipt is not received within 2 working days contact the Data Manager / Senior Trial Co-ordinator at CTRU***.

#### Reporting pregnancies of partners

Pregnancies of partners of trial participants should be reported using the Pregnancy Reporting CRF and faxed to the CTRU within 24 hours of becoming aware of the pregnancy.

Fax: CTRU: 0113 343 7985

A receipt will be sent for all received Pregnancy Reporting CRFs. ***If a receipt is not received within 2 working days contact the Data Manager/Senior Trial Co-ordinator at CTRU***.

# CONTENTS

1. [KEY CONTACTS 2](#_bookmark0)
2. [CONTENTS 8](#_bookmark1)
3. [TRIAL FLOW DIAGRAM 11](#_bookmark2)
4. [BACKGROUND 12](#_bookmark3)
   1. [Clinical and scientific relevance 12](#_bookmark4)
   2. [Potential benefits to participants and the NHS 12](#_bookmark5)
5. [AIMS AND OBJECTIVES 13](#_bookmark6)
   1. [Primary Objectives 13](#_bookmark7)
   2. [Secondary Objectives 13](#_bookmark8)
6. [DESIGN 13](#_bookmark9)
7. [ELIGIBILITY 14](#_bookmark10)
   1. [Inclusion criteria 14](#_bookmark11)
   2. [Exclusion criteria 14](#_bookmark12)
8. [RECRUITMENT, REGISTRATION AND RANDOMISATION 15](#_bookmark13)
   1. [Recruitment 15](#_bookmark14)
   2. [Informed consent and eligibility 15](#_bookmark15)
      1. [Optional Preliminary Consent for Eligibility Assessment 16](#_bookmark16)
      2. [Loss of Capacity Following Informed Consent 17](#_bookmark17)
   3. [Non-Registration 17](#_bookmark18)
   4. [Registration 17](#_bookmark19)
   5. [Randomisation 19](#_bookmark20)
9. [TRIAL MEDICINAL PRODUCT MANAGEMENT 20](#_bookmark21)
   1. [Supply of Tostran and placebo 20](#_bookmark22)

[9.1.2 Dispensing of Tostran and placebo 20](#_bookmark23)

- 1. [Storage 21](#_bookmark24)
  2. [Distribution 21](#_bookmark25)
  3. [Packaging and labelling 21](#_bookmark26)
     1. [Tostran 2% Gel and Placebo (blinded) 21](#_bookmark27)
  4. [Blinding 22](#_bookmark28)
  5. [Recording compliance with treatment 22](#_bookmark29)
  6. [Destruction of canisters 22](#_bookmark30)
  7. [Replacing stock 23](#_bookmark31)

1. [TREATMENT DETAILS 23](#_bookmark32)
   1. [Treatment Regimen Details 23](#_bookmark33)
   2. [Administration of Tostran/placebo 23](#_bookmark34)
   3. [Compliance 24](#_bookmark35)
   4. [Titration 24](#_bookmark36)
   5. [Emergency Unblinding 25](#_bookmark37)
   6. [Treatment of participants following emergency unblinding 26](#_bookmark38)
   7. [Withdrawal of treatment 26](#_bookmark39)
   8. [Unblinding of participants at the end of treatment 26](#_bookmark40)
2. [ASSESSMENTS / SAMPLES / DATA COLLECTION 27](#_bookmark41)
   1. [Schedule of events 28](#_bookmark42)
   2. [Assessment of eligibility, registration and randomisation 29](#_bookmark43)
   3. [Treatment Assessments 29](#_bookmark44)
      1. [Assessments required at baseline 29](#_bookmark45)
      2. [Assessments required after 2 weeks of treatment (+/- 2 days) 30](#_bookmark46)
      3. [Telephone contact after 6 (± 1) weeks of randomisation 30](#_bookmark47)
      4. [Assessments required after 13 (± 1) weeks of randomisation 31](#_bookmark48)
      5. [Telephone contact after 19 (± 1) weeks of randomisation 31](#_bookmark49)
      6. [Assessments required after 26 (± 1) weeks of randomisation 31](#_bookmark50)
      7. [Telephone contact 30 days after finishing treatment 32](#_bookmark51)
   4. [Sample Handling 32](#_bookmark52)
   5. [Adverse and Serious Adverse Events 32](#_bookmark53)
   6. [Reporting of pregnancies 33](#_bookmark54)
   7. [Deaths 33](#_bookmark55)
   8. [Definition of the end of trial 33](#_bookmark56)
   9. [Central review of DXA scans 33](#_bookmark57)
3. [PHARMACOVIGILANCE PROCEDURES 33](#_bookmark58)
   1. [General definitions 33](#_bookmark59)
      1. [Adverse Events (AEs) 33](#_bookmark60)
      2. [Serious Adverse Events (SAEs) 33](#_bookmark61)
      3. [Suspected Unexpected Serious Adverse Reactions (SUSARs) 34](#_bookmark62)
   2. [Operational definition and reporting AEs 34](#_bookmark63)
      1. [Expected AEs 34](#_bookmark64)
      2. [Unexpected AEs 35](#_bookmark65)
   3. [Operation definition – Serious Adverse Events 35](#_bookmark66)
      1. [Expected SAEs 35](#_bookmark67)
   4. [Recording and reporting SAEs, SSARs and SUSARs 35](#_bookmark68)
   5. [Responsibilities 36](#_bookmark69)
4. [QUALITY OF LIFE (QOL) 37](#_bookmark70)
   1. [Timing and administration of quality of life questionnaires 38](#_bookmark71)
5. [ENDPOINTS 38](#_bookmark72)
   1. [Primary endpoints 38](#_bookmark73)
   2. [Secondary endpoints 39](#_bookmark74)
6. [STATISTICAL CONSIDERATIONS 39](#_bookmark75)
   1. [Sample size 39](#_bookmark76)
   2. [Planned recruitment rate 40](#_bookmark77)
7. [STATISTICAL ANALYSIS 41](#_bookmark78)
   1. [General considerations 41](#_bookmark79)
   2. [Frequency of analyses 41](#_bookmark80)
   3. [Endpoint analyses 41](#_bookmark81)
8. [DATA MONITORING 42](#_bookmark82)
   1. [Data Monitoring and Ethics Committee 42](#_bookmark83)
   2. [Data Monitoring 42](#_bookmark84)
   3. [Clinical governance issues 42](#_bookmark85)
9. [QUALITY ASSURANCE AND ETHICAL CONSIDERATIONS 42](#_bookmark86)
   1. [Quality Assurance 43](#_bookmark87)
   2. [Ethical considerations 43](#_bookmark88)
10. [CONFIDENTIALITY 43](#_bookmark89)
11. [ARCHIVING 44](#_bookmark90)
12. [STATEMENT OF INDEMNITY 44](#_bookmark91)
13. [STUDY ORGANISATIONAL STRUCTURE 44](#_bookmark92)
    1. [Responsibilities 44](#_bookmark93)
    2. [Operational Structure 45](#_bookmark94)
14. [PUBLICATION POLICY 46](#_bookmark95)
15. [KEY REFERENCES 47](#_bookmark96)

[APPENDIX A - GLOSSARY OF TERMS 49](#_bookmark97)

[APPENDIX B: Clinical Trial Risk Assessment Document 51](#_bookmark98)

[What are the key risks related to therapeutic interventions you plan to monitor in this trial? .. 51](#_bookmark99) [How will these risks be minimised? 51](#_bookmark100)

[IMP/Intervention 51](#_bookmark101)

[Body system/Hazard 51](#_bookmark102)

[Activity 51](#_bookmark103)

[Frequency 51](#_bookmark104)

[Comments 51](#_bookmark105)

# TRIAL FLOW DIAGRAM

Screening

Written informed consent

Registration

Eligibility confirmation

(inc. measurement of serum testosterone level)

Baseline Assessments and Randomisation

Tostran 2% testosterone gel 3g gel/day (containing 60mg testosterone)

Placebo gel

3g gel/day (containing no active substance)

2 week Dose titration

Dose adjusted based on participant’s serum testosterone levels (analysed centrally)

2 week Dose titration

Dose adjustment allocated at random to maintain blinding

6 Week Follow-up by telephone

13 Week Follow-up Assessment in clinic

19 Week Follow-up by telephone

26 Week Follow-up Assessment in clinic

Follow-up by telephone 30 days after the end of treatment

# BACKGROUND

## Clinical and scientific relevance

Approximately 1% of UK men aged 25-50 are cancer survivors1. Our recent study of 176 men in Sheffield and Manchester2 demonstrated that 27% of male cancers survivors aged 25-45 have serum testosterone below the 10th centile for age-matched controls (<12nmol/l), and half of these have serum testosterone below the 2.5th centile (<10nmol/l). Cancer survivors had higher fat mass and fasting insulin:glucose ratio, and worse quality of life scores than controls (particularly physical functioning, vitality and sexual function)3. Survivors had on average 2.2kg greater truncal fat mass and 2nmol/l lower testosterone and the evidence suggested the low testosterone was causative for the increased fat mass. Alterations in testosterone and body composition may have major effects on health; low testosterone and high truncal fat are associated with increased risk of vascular disease4 and diabetes5 (independently of Body Mass Index (BMI)) and waist circumference is associated with all-cause mortality, even in subjects with a normal BMI6. As cancer treatments improve, the number of cancer survivors is increasing; we estimate that currently around 1 in 450 men aged 25- 50 will be a survivor of lymphoma, testicular cancer or leukaemia with low testosterone.

Previous studies confirm that treatment for testicular cancer, lymphoma and leukaemia can result in hypogonadism7-9, increased BMI10, increased truncal fat mass11 and impaired quality of life scores12. Testosterone replacement in older men improves body composition, insulin sensitivity, mood, fatigue and sexual functioning13-16. In younger hypogonadal men testosterone replacement increases lean mass, but the effects on insulin sensitivity may be less marked17,18. The only previous study of testosterone replacement in male cancer survivors demonstrated a small improvement in fatigue, but no change in body composition, mood or sexual function19. However, the number of subjects was small (35 subjects in total) and most had serum testosterone levels in the normal range, with a relatively small increase on treatment.

It is well recognised that cancer and its treatment are associated with long term physical and psychological effects20, many of which are independent of gonadal function, and male cancer survivors with normal testosterone have lower quality of life scores than controls7,21. Increased fat mass and insulin resistance are likely to have long-term effects on cardiovascular risk. A recent informal patient survey conducted for the first meeting of the survivorship group of the National Cancer Research Insitute (NCRI) Testis Clinical Studies Group (CSG) identified fatigue, sexual function and reintegration into work as areas of concern for patients. It is unknown how much of the adverse body composition and quality of life scores in hypogonadal men is reversible with testosterone replacement. A randomised controlled trial is therefore needed to address this patient- identified consequence of their cancer treatment and for clinicians to have a sound evidence-base to treatment where there is currently a knowledge gap.

## Potential benefits to participants and the NHS

Fatigue is a common and distressing problem for cancer patients, and can impair ability to return to employment after treatment. Sexual dysfunction can have significant effects on relationships and

quality of life. Adverse body composition and insulin resistance are associated with increased risk of diabetes and vascular events.

All of these problems impact on individual patients and National Health Service (NHS) resources. Testosterone gel is a safe, well-tolerated and inexpensive therapy. If we demonstrate significant benefits in this patient group, we may be able to improve their quality of life, reduce their vascular risk and decrease their need for NHS services in the future.

# AIMS AND OBJECTIVES

This trial aims to establish whether testosterone replacement therapy can improve body composition and quality of life in young hypogonadal male cancer survivors.

## Primary Objectives

- - - To assess the effect of 26 weeks of treatment with testosterone gel on truncal fat mass in hypogonadal cancer survivors
    - To assess the effect of 26 weeks of treatment with testosterone gel on participant self- reported physical function scores in hypogonadal cancer survivors

## Secondary Objectives

- - - To assess the effect of 26 weeks of treatment with testosterone gel on lean body mass, whole body fat mass, BMI, waist circumference, blood insulin, glucose, lipid and luteinizing hormone (LH) levels and bone density in hypogonadal cancer survivors
    - To assess the effect of 26 weeks of treatment with testosterone gel on participant self- reported quality of life, fatigue, self-esteem and sexual function scores in hypogonadal cancer survivors

# DESIGN

TRYMS is a prospective, multicentre, randomised, doubled-blinded, parallel group, placebo controlled phase IV superiority clinical trial in young male cancer survivors with low testosterone levels. A parallel group design rather than a cross-over study was chosen as the length of the effects of testosterone on body composition are unknown and, for this reason, it would not be possible to ensure a sufficient wash-out period. The treatment period will be for 26 weeks.

The trial is designed to investigate whether testosterone treatment will result in a reduction of truncal fat mass and an increase in participant self-reported physical functioning scores.

A minimum of 112 (up to a maximum of 268) eligible participants will be randomised on an equal basis to receive either Tostran 2% (testosterone) Gel or the Tostran gel placebo.

The double-blind trial design in which participants and site personnel will be blinded to the treatment allocation will minimise the possible induction of bias.

At 2 weeks into treatment serum testosterone levels will be measured 2 hours after applying the gel and a dose titration performed. Further information regarding the dose titration can be found in section 10.4.

All participants will be followed up for a period of 26 weeks with an additional safety follow-up telephone call 30 days after finishing treatment. (Please refer to section 11 on Assessments/samples/data collection for more details on the follow-up schedule).

# ELIGIBILITY

Eligibility waivers to the inclusion and exclusion criteria are not permitted.

## Inclusion criteria

Participants with the following characteristics are eligible for this trial:

- - - Male
    - Aged between 25 and 50 years
    - Post-pubertal
    - Previous testicular cancer, lymphoma or leukaemia
    - A serum testosterone level ≥7nmol/l and ≤12nmol/l
    - At least 12 months from completion of curative treatment for testicular cancer (curative surgery without chemotherapy is permissible), lymphoma or leukaemia
    - If had allogeneic bone marrow transplant more than 12 months from end of glucocorticoid treatment
    - If taking any hormone replacement, on stable doses for the last 6 months
    - Able and willing to provide written informed consent
    - Able and willing to comply with all study procedures
    - Able to start treatment within 2 days of randomisation

## Exclusion criteria

- Body Mass Index (BMI) of more than 35 kg/m2
- Currently receiving oral or IV corticosteroid therapy or likely to receive corticosteroids during the trial
- Previous testosterone treatment within 12 months of entering the trial
- A history of allergic reaction to Tostran gel
- Active chronic graft vs host disease
- A history of hormone-dependent cancer (e.g. prostate or breast cancer)
- A history of primary liver tumour
- Disease or current medication known to have significant effects on body fat mass or distribution (e.g. inherited lipodystrophy or HIV drugs)
- Nephrotic syndrome
- Type 1 diabetes mellitus (patients with type 2 diabetes may be included if they have been on stable treatment for six months)
- Moderate to severe heart failure
- Severe obstructive sleep apnoea
- Active liver disease (fatty liver is not an exclusion)
- Uncontrolled hypertension: blood pressure > 100 mmHg diastolic or > 160 mmHg systolic after resting and three measurements (repeat measurement is not necessary if the first value is below 160/100)
- Renal impairment; estimated Glomerular Filtration Rate (eGFR) <30ml/min
- Clinically significant abnormal Prostate-Specific Antigen (PSA) or Full Blood Count (FBC)
- Hypercalcaemia
- Other severe concurrent disease or mental disorder which would affect the collection of study measurements
- If intending on trying to conceive in the next 12 months (unless intending to use previously banked sperm)

It is recommended that patients receiving oral anticoagulants have their International Normalised Ratio (INR) closely monitored especially when the treatment is started, stopped or the dose changed.

Participants who have been randomised into the trial may not be randomised again for any reason.

# RECRUITMENT, REGISTRATION AND RANDOMISATION

## Recruitment

Participants will be recruited from multiple research sites around the United Kingdom. Research sites will be required to have obtained management approval and undertake a site initiation meeting with the CTRU prior to the start of recruitment into the trial.

The recruitment target requires that a minimum of 112 and a maximum of 268 participants are recruited in to the trial over a 3 year period.

## Informed consent and eligibility

Potential participants will be identified from clinic lists based on the inclusion criteria of age and cancer diagnosis.

Participants will be approached during standard clinic visits for management of their disease and will be provided with verbal and written details about the trial. Clinic patients may also be approached by invitation letter from their usual care team with a follow-up phone call from the study team to ask if they would be willing to receive further information about the study.

Participants will also be identified through disease-specific cancer databases, and approached by written invitation. Participants that are identified through cancer databases should be approached through their GP using a standard letter of invitation provided by the CTRU.

All participants who agree to receive further information about the study will be provided with a Participant Summary Sheet containing a concise summary of the study and a Participant Information Sheet, including detailed information about the rationale, design and personal implications of the trial. Participants identified through disease-specific cancer databases and approached through their GP and those clinic patients approached by invitation letter will also be provided with an ‘Invitation to be Part of Cancer Research’ document containing a concise non- specific summary of clinical trials. Following information provision, participants will have as long as they need to consider participation in the trial (normally a minimum of 24 hours) and will be

given the opportunity to discuss the trial with their family and other healthcare professionals before they are asked whether they would be willing to take part in the trial.

Assenting participants will be broadly assessed for eligibility during the screening process based on their medical history according to the inclusion and exclusion criteria.

Potential participants will be asked to provide written informed consent. The Principal Investigator or any other doctor who has received Good Clinical Practice (GCP) training and is authorised on the trial delegation log is permitted to take informed consent for trial participation. The right of the participant to refuse consent without giving reasons will be respected. Further, the participant will be told that they are free to withdraw from the trial at any time without giving reasons and without prejudicing any further treatment.

A record of the consent process detailing the date of consent and all those present will be kept in the participants’ notes. The original consent form(s) will be filed in the Investigator Site File, a copy of the consent form(s) will be given to the participant and a copy of the consent form(s) will be returned to the Clinical Trials Research Unit (CTRU), at the University of Leeds.

After written informed consent, participants will be registered on the trial and blood will be taken to confirm eligibility based on serum testosterone and safety screening bloods.

When all eligibility criteria are confirmed, the participant can be randomised. Participants who consented to the eligibility assessments using the Optional Preliminary Participant Information Sheet and Informed Consent Document for Eligibility (see section 8.2.1) must also consent using the full Participant Information Sheet and Informed Consent Document for the trial prior to randomisation.

Participants who consent but are ineligible may be re-consented and re-screened after 6 months if the reason for their ineligibility was temporary or if their serum testosterone was close to the eligible range for the study. Participants who are re-screened will not be re-registered and will keep their original trial number.

### Optional Preliminary Consent for Eligibility Assessment

To reduce additional visits and inconvenience for patients, a preliminary consent to the eligibility assessments can be obtained at the initial approach in clinic so that bloods can be taken immediately (if between 8 am and 10 am (+/-1 hour)). This can be done using the Optional Preliminary Participant Information Sheet and Informed Consent Document for Eligibility. The optional preliminary consent may be taken by an appropriately qualified member of the trial team (including nurses and other health care professionals) who has received GCP training and is authorised on the trial delegation log to take this consent.

If the participant is found to be eligible, would like to take part in the study and has already been provided with the full Participant Information Sheet for the trial, at the next visit he can be consented by a doctor who has received GCP training and is authorised on the trial delegation log (using the full Participant Information Sheet and Consent Document) and randomised (which must be within 35 days of the eligibility blood sample being taken). If a participant has any concerns when attending clinic and requires more time to consider, more time can be offered prior to full consent being taken but a repeat blood sample will be required if randomisation does not take place within 35 days of the eligibility blood sample being taken.

The optional preliminary consent is purely for the purpose of carrying out eligibility assessments including a blood sample taken between 8 am and 10 am (+/- 1 hour) to measure serum testosterone and assess safety for randomisation (see section 11.2 for details of eligibility assessments). Participants who provide the optional preliminary consent are not consenting to participate in the trial therefore trial-specific assessments not relating to eligibility (see section 11.3) can only be carried out following full consent for the trial using the Participant Information Sheet and Consent Document.

All participants who consent (either optional preliminary consent or full consent) will be registered into the trial.

### Loss of Capacity Following Informed Consent

Participants who lose mental capacity after informed consent has been obtained will be withdrawn from trial treatment and follow-up; any ongoing Adverse Events (AEs) must still be collected for 30 days.

## Non-Registration

Each trial research site will be required to maintain an ongoing log of all participants considered for the trial who are not registered either due to ineligibility or because they declined participation. Anonymised information will be collected including:

- Age
- Ethnicity
- Reason for ineligibility for trial participation/reason for declining participation despite being eligible

Non-registration logs will be returned to the CTRU by trial research sites on a regular basis, at least quarterly.

## Registration

**Timing of registration and randomisation**

Recruitment of participants to the TRYMS trial requires a trial-specific investigation to confirm eligibility. Therefore, recruitment is a two-step process involving consent of all potential participants prior to conducting investigations to confirm eligibility. All participants who are consented must be registered into the trial. Participants are randomised into the trial within 35 days of the date that the eligibility blood sample was taken. The order of events between consent and the start of trial treatment is shown in Figure 1.

N

Figure 1: Schedule of events between consent and start of trial treatment


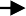

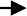

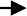

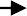
≤35 days

Consent

*(Full or optional preliminary)*

Registration

Eligibility:

Medical history Height & weight Blood pressure

Blood sample (8-10am, +/- 1 hour)*

Ensure full consent has been taken before randomisation

Randomisation

Treatment starts

≤2 days

Y

- The blood sample for confirmation of eligibility must be taken after consent but may be taken before registration if necessary. All participants who consent must be registered.

Baseline assessments:

(on day of randomisation where possible)

Quality of Life Waist circumference Whole body DXA Fasted blood sample

Serum samples stored for later central measurement (8-10am, +/- 1 hour) Concomitant medications

End of participation

Following written informed consent participants will be registered in to the trial by an authorised member of staff at the trial research site. **Participants must have consented before any trial specific procedures are performed.** Sites where total testosterone, FBC, Urea and Electrolytes (U+E), Liver Function Test (LFT), calcium and PSA are measured as part of standard practice should provide written confirmation of this standard practice if the results of standard investigations are used for eligibility purposes and trial-specific consent is not obtained prior to the blood sample being taken.

#### All participants who consent (optional preliminary consent or full consent) must be registered into the trial.

Registration will be performed centrally using the CTRU automated 24-hour telephone service. Authorisation codes and PINs, provided by the CTRU after site initiation, will be required to access the 24-hour telephone service.

Please complete Form 01 – Registration prior to phoning the 24-hour telephone service. The following information will be required at registration:

- - Site code
  - Name of person making the registration
  - Confirmation of written informed consent
  - Participant details, including participant initials and date of birth.

Following registration participants will receive a trial number.

Direct line for 24-hour registration 0113 343 8265

Once the participant’s eligibility for randomisation has been confirmed they can be randomised. Eligible participants who consented using the optional preliminary consent form for the eligibility assessment must also consent using the full consent form for the trial prior to randomisation.

## Randomisation

Following registration, confirmation of eligibility for randomisation and provision of full consent, participants will be randomised by an authorised member of staff at the trial research site. Participants should be randomised no later than 35 days after the date that the eligibility blood sample was taken. Randomisation will be performed centrally using the CTRU’s automated 24-hour telephone service. Authorisation codes and PINs, provided by the CTRU after site initiation, will be required to access the 24-hour telephone service.

Please complete Form 03 – Randomisation prior to phoning the 24-hour service. The following information will be required at randomisation:

- Site code
- Name of person making the randomisation
- Trial number
- Participant date of birth
- Confirmation of eligibility
- Date and time of testosterone sample
- Stratification factors (see list below)

Direct line for 24-hour randomisation 0113 343 8265

A computer-generated minimisation programme that incorporates a random element will be used to ensure treatment groups are well-balanced for the following participant characteristics, details of which will be required for randomisation:

- Serum testosterone level (7-9.9 nmol/l, 10-12 nmol/l)
- Type of previous cancer (testicular cancer, lymphoma, leukaemia)

• Body Mass Index (BMI) (<25 kg/m2, 25-29.9 kg/m2, 30-35 kg/m2)

- Age (25-37 years, 38-50 years)
- Time between end of curative anti-cancer treatment and study entry (12-30 months, 31-60 months, 61+ months)
- Randomising site

Participants will be randomised on a 1:1 basis to receive either:

- Tostran 2% (testosterone) gel
- Tostran gel placebo

At the end of this phone call the participant’s randomisation allocation will not be disclosed in order to maintain the blinding of the trial. Instead, a unique kit code will be provided which identifies a box of canisters that need to be dispensed by pharmacy. The CTRU will send a fax notification to pharmacy to inform them of the randomisation and which box of canisters needs to be provided to the participant. This fax is generated and sent automatically. In the event of a system failure, the kit code may need to be provided to the pharmacy directly by the member of site staff randomising the participant.

# TRIAL MEDICINAL PRODUCT MANAGEMENT

Please refer to the TRYMS Pharmacy and Investigational Medicinal Product (IMP) Study Site Operating Procedure (SSOP) for full details of the trial IMP management requirements. Within the trial the following are classed as IMPs:

#### Tostran 2% Gel

- One gram of gel contains 20mg testosterone. One press of the canister piston delivers 0.5g of gel containing 10mg testosterone.
- Supplied by ProStrakan
- Please refer to the Summary of Product Characteristics (SmPC) for Tostran 2% Gel in accordance with local procedures

#### Placebo Gel

- Composition: Propylene glycol, ethanol anhydrous, isopropylalcohol, oleic acid, carbomer 1382, trolamine, butylhydroxytoluene (E321), water, purified and hydrochloric acid (for pH) adjustment
- Supplied by ProStrakan

## Supply of Tostran and placebo

Tostran 2% Gel and placebo gel will be provided free of charge by ProStrakan for use in this clinical trial. Blinded supplies will be sent to trial research sites in pre-labelled canisters which will be identifiable by a kit code printed on to the label. Canisters will be packaged in to a box containing 6 canisters; one box will be dispensed to a participant at a time and will be used for 13 weeks of treatment. The 6 canisters in each box and the outer packaging will all be identifiable by the same unique kit code.

Tostran 2% Gel and placebo will be distributed in accordance with the TRYMS Pharmacy and IMP SSOP provided within the Pharmacy Site File.

#### The supply of placebo and Tostran must not be used for any purpose other than that outlined in this protocol and should be clearly ring-fenced from standard hospital stock.

### 9.1.2 Dispensing of Tostran and placebo

The relevant site pharmacist will be notified by fax from the CTRU of all participants randomised at that site; the fax notification will also detail which box of canisters should be given to the participant. The person randomising the participant will also have been told which kit code should

be dispensed whilst making the randomisation telephone call. In order to maintain the blinding of the trial the pharmacist will not be told the participants treatment allocation.

Blinded canisters will be dispensed to participants at the start of treatment and again at week 13. At the dispensing visits at baseline and week 13 the participants will receive one box, containing 6 canisters, of either Tostran or placebo, identifiable only by the unique kit code on both the outer box and the canisters contained within the box.

**At week 13, site staff must call the 24-hour telephone system again before dispensing stock in order to obtain the kit code of the second box of canisters to dispense to the participant.** This should be done by an authorised member of site staff as delegated on the Authorised Personnel Log. This will generate a further fax from CTRU to pharmacy to inform them of the kit code of the correct box of canisters to dispense.

#### Each box of canisters will have a corresponding Emergency Unblinding Envelope. Each time a box of canisters is dispensed the participant’s trial number must be added to the corresponding Emergency Unblinding Envelope and held securely within the pharmacy.

All IMP stock received and dispensed should be recorded on the Accountability and Dispensing Logs.

## Storage

Canisters should be stored upright at room temperature and in the original packaging. If the storage temperature exceeds 25°C please quarantine the stock and contact CTRU. All canisters supplied for use within the trial should be stored in a secure ring-fenced location within pharmacy.

## Distribution

Tostran and placebo gel for use within the trial will be sent from ProStrakan to the Pharmacy Department of University College London Hospitals NHS Foundation Trust which holds a Manufacturer’s Authorisation for Investigational Medicinal Products. Here the canisters and boxes will be labelled in accordance with Directive 2001/20/EC and the Medicines for Human Use (Clinical Trials) Regulations 2004 (as amended) to maintain the blinding of the trial.

Blinded canisters will be sent from University College London Hospitals NHS Foundation Trust, packaged in to boxes of 6, to Sheffield Teaching Hospitals NHS Foundation Trust for distribution to sites.

## Packaging and labelling

### 9.4.1 Tostran 2% Gel and Placebo (blinded)

Tostran and the placebo gel will be shipped to sites in pre-labelled canisters. In order to maintain the blinding of the trial these canisters will be identical and both labelled with the same study specific label in accordance with Directive 2001/20/EC and the Medicines for Human Use (Clinical Trials) Regulations 2004 (as amended). The canisters will be identifiable by a unique kit code printed on to the label. To ensure that the correct treatment is dispensed to the participant the relevant site pharmacist will be told which canisters to dispense to each participant using this numbering system.

The participant’s trial number should be added to the label on each canister and the outer box by the pharmacist or authorised delegate at the time of dispensing.

## Blinding

The following controls will be employed to maintain the double blind status of the trial:

- - - The Tostran and placebo gels and canisters will be identical in appearance and have the same labelling.
    - Canisters will be identified by a unique kit code assigned at random. The CTRU trial statistician will be responsible for maintaining this list, which will be securely password protected when treatment information is contained within the list.
    - The Investigator and other members of the site staff involved with the trial and the participants themselves will remain blinded to the treatment allocation (except where emergency unblinding is necessitated).

The following outline how unblinding will be kept to a minimum for tasks to be conducted unblinded at the CTRU:

- - - Management of kit-codes on the kit logistics application which is linked to the 24-hour randomisation system will be conducted by the Trial Statistician in addition to maintaining the back-up kit-code lists for each centre.
    - Any unblinded interim reports provided to the Data Monitoring and Ethics Committee (DMEC) will be provided by the CTRU Trial Statistician and the reports will be securely password-protected.
    - Suspected Unexpected Serious Adverse Events (SUSARs) will be unblinded by the CTRU trial statistician. If the event requires expedited reporting, the Trial Statistician will pass the SUSAR to an unblinded independent CTRU staff member responsible for electronic SUSAR reporting to the Medicine and Healthcare Regulatory Agency (MHRA) and the ethics committee. Unblinded records of SUSARs reported will be maintained by the statistician.
    - The results of serum testosterone measurements received at the CTRU will be managed by unblinded data management CTRU staff who are independent to the trial team (except in the case of two week titration results which are out-of-range or cases where repeat samples are required, which will be handled by the trial Data Manager due to the complexity of this process).

Code break procedures are outlined in section 10.5 and described in detail in the Emergency Unblinding SSOP provided in the Investigator Site File and Pharmacy Site File.

## Recording compliance with treatment

In order to record compliance with treatment all used canisters will be returned to pharmacy and weighed to calculate how much gel has been used from each canister. Canisters will be returned to pharmacy at weeks 13 and 26. The canisters should be weighed without the lid and the weight recorded in grams to 1 decimal place. This information should be recorded on the Returned Canisters Log within the Pharmacy Site File. The scales that are used to weigh canisters should be calibrated and a record kept of the calibration.

## Destruction of canisters

Used canisters should only be destroyed after they have been weighed, in order to record compliance, as detailed above. Once they have been weighed they can be destroyed as per each

research site’s local policy. Canisters of expired or unstable stock should be quarantined and retained until destruction is authorised by the CTRU.

## Replacing stock

If a participant requires any replacement stock in between visits it is possible to have stock replaced using the CTRU 24-hour telephone service. Before telephoning to get stock replaced please complete the Replacement Stock Case Report Form (CRF) which can be found within the Investigator Site File which also contains further instructions.

# TREATMENT DETAILS

The following section of the protocol describes treatment for participants with Tostran/placebo.

## Treatment Regimen Details

Participants will be randomised to receive either Tostran or placebo gel for 26 weeks. Participants will require IMP to be dispensed at **baseline** and **13 weeks**. When dispensing IMP at baseline and 13 weeks the CTRU 24-hour telephone service should be phoned and this will inform the site of the kit code for the box of canisters to dispense. Pharmacy will receive a fax at baseline and Week 13 to inform them which kit code to use.

## Administration of Tostran/placebo

Participants will receive Tostran or placebo gel for cutaneous use. All participants should apply 3 grams of gel (6 pumps of the canister) once a day for the first two weeks of treatment. After two weeks of treatment serum testosterone levels will be measured centrally by Sheffield Teaching Hospitals NHS Foundation Trust, at the Royal Hallamshire Hospital. For those participants on active treatment their dose will be adjusted based on their testosterone level. In order to maintain the blinding of the trial, participants receiving placebo will have their dose adjusted at random.

The dose adjustment will ensure that each participant receiving Tostran is receiving the optimum dose. All participants will be informed by the research team at site by telephone and in writing of the dose to take for the remainder of the trial. Further information regarding dose titration is provided in section 10.4. Participants should continue using the canisters given to them at baseline and just adjust the number of pumps as instructed.

Each full depression of the canister piston delivers 0.5g of gel (containing 10mg of testosterone for participants receiving Tostran). The table below shows the amount of gel dispensed and the amount of testosterone which would be applied to the skin from a number of piston depressions.

| Number of Depressions | Amount of Gel (g) | Amount of Testosterone Applied to the Skin (mg) |
| --- | --- | --- |
| 1 | 0.5 | 10 |
| 2 | 1 | 20 |
| 4 | 2 | 40 |
| 6 | 3 | 60 |
| 8 | 4 | 80 |

Before obtaining the first full dose the canister should be primed by fully depressing the actuator 8 times; this will be done at home by the participant. The gel that is dispensed during priming should

TRYMS_V6.8_14_06Jul_04_SponsorID STH15216

Page 23 of 52

be discarded; it is only necessary to prime the canister before the first dose. The canister should be stored at room temperature in an upright position between uses.

The dose can be applied to the abdomen (entire dose over an area of at least 10 by 30 cm) or to **both** inner thighs (one half of the dose over an area of at least 10 by 15cm for each inner thigh). Daily rotation between the abdomen and inner thighs is recommended to minimise application site reactions. Tostran/placebo gel should not be applied to the genitals.

The gel should be applied to clean, dry intact skin. It should be rubbed in gently with one finger until dry, and then the application site should be covered, preferable with loose clothing. Hands should then be washed with soap and water. The gel should be applied in the morning. Participants who wash in the morning should apply the gel after washing, bathing or showering.

## Compliance

In order to assess participant compliance with the trial treatment, used canisters must be returned to the hospital pharmacy at weeks 13 and 26 for weighing in grams (to 1 decimal place). Canisters should be weighed without the lid.

## Titration

Serum testosterone concentration will be measured two weeks (no less than 12 days) after starting trial treatment. The blood sample for measuring serum testosterone level should be obtained 2 hours after application of treatment (up to 4 hours after application of gel will be allowed). The participant should wash the phlebotomy area with soap and water prior to the blood sample being taken to avoid potential contamination of the sample. Serum samples will be sent to the Royal Hallamshire Hospital in Sheffield for central measurement, and the results will be communicated directly to the CTRU. An additional retest may be required within four weeks of the first titration test for reasons such as; timings of samples are not as per the protocol, an error in the central measurement has occurred, testosterone levels are outside of the range 5 to 40 nmol/l or to maintain the blind.

The results from these samples will be entered in to the CTRU 24-hour telephone system which will calculate the recommended future dose based on the current testosterone levels, for participants receiving active treatment. For participants receiving placebo the recommendation will be generated at random.

For participants who are receiving Tostran and whose testosterone is within the range of 5 to 40 nmol/l their recommended ongoing dose will be based on their serum testosterone value as detailed in the table below. The dose adjustments will be sent to sites by fax and will not contain any information related to the participant’s trial arm or testosterone value. **This recommendation will not be reviewed by a medically trained clinician at the CTRU and as such all recommendations should be reviewed by a medically qualified member of the trial team at site to ensure that the amended dose is clinically appropriate.** Those participants requiring a retest should continue to apply 3 grams of gel (6 pumps of the canister) once a day until notified of the amended dose.

All participants whose testosterone levels are outside of the range 5 to 40 nmol/l will have their results reviewed by an unblinded endocrinologist who is independent to the trial team to identify any safety issues. This may result in the treating clinician and participant being unblinded and having to stop trial treatment.

If serum testosterone is lower than 11nmol/l the daily dose will be increased by 20mg, if serum testosterone is between 11 and 15nmol/l the daily dose will be increased by 10mg. If the

testosterone level is greater than 15 but lower than 35 the daily dose will remain the same and if the level is between 35nmol and 40nmol/l then the daily dose will be reduced by 20mg. If the testosterone level is greater than 40nmol/l the unblinded endocrinologist will determine an appropriate dose within the range specified in the table below:

| **Serum testosterone level** | **Amended dose** |
| --- | --- |
| < 11 nmol/l | 80mg/day |
| 11 – 15 nmol/l | 70mg/day |
| > 15 - < 35nmol/l | 60mg/day (no change) |
| 35nmol/l - 40nmol/l | 40mg/day |
| >40nmol/l | 40-20mg/day |

Participants randomised to receive placebo will be assigned to increase dose, decrease dose or stay at the same dose by the CTRU 24 hour telephone system using a pre-defined randomly generated list to maintain blinding of investigators and participants, as detailed above.

Site will be informed of the participants’ planned dose adjustment by fax. This will take approximately 10 days from the time of the sample being received. Participants should continue to use 3 grams of gel during this time. The dose recommendation should be reviewed by a medically qualified person to confirm that the suggested dose adjustment is clinically appropriate. It is the clinician’s responsibility to inform CTRU as soon as possible if the dose recommendation is considered clinically inappropriate. This may result in the participant being unblinded.

Participants will be informed by telephone and in writing of their adjusted dose by an appropriate member of site staff. This adjusted dose should be used for the remainder of the treatment period.

An additional serum testosterone measurement will be performed at 26 weeks after randomisation. Samples taken at baseline and 26 weeks will be stored locally until the end of the trial when they will be sent to the Royal Hallamshire Hospital in Sheffield for central analysis. These samples should not be assessed locally. See section 11.4 for further information about Sample Handling.

## Emergency Unblinding

The only approved reason for emergency unblinding a participant’s treatment allocation is when information about the participant’s trial treatment is clearly necessary, and will alter, the appropriate medical management of the participant.

The unblinding process will be undertaken by telephoning the CTRU during office hours (9.00 to

- 1. Monday to Friday excluding public/bank holidays, the period between Christmas and New Year and all Tuesdays following a bank holiday except for Mayday). The person telephoning to request an emergency unblinding should have the following information available at the time of telephoning:
     - Participant details, including trial number, initials and date of birth
     - Name of trial research site and site code
     - Name of person making the request for an emergency unblinding
     - Reason for requesting an emergency unblinding

Following the emergency unblinding of a participant, the CTRU Data Manager will send a notification to the Principal Investigator and the local pharmacy. The details of the emergency unblinding should be recorded on the Unblinding Log provided.

**Direct line for CTRU emergency unblinding: 0113 343 4930**

Outside of office hours, or where the Investigator is unable to contact CTRU, emergency unblinding may also be undertaken by the local pharmacist. Unblinding envelopes will be provided to pharmacy at the time of IMP delivery and each envelope will be linked to a specific box of canisters using a unique kit code. If an emergency unblinding occurs at site the CTRU should be notified immediately by fax. Instructions for doing this are contained within the unblinding envelope.

Further information on emergency unblinding can be found in the Emergency Unblinding SSOP located within the Investigator Site File and Pharmacy Site File.

## Treatment of participants following emergency unblinding

Following an emergency unblinding the participant should stop trial treatment and be treated in line with local hospital policy.

## Withdrawal of treatment

In line with usual clinical care, cessation or alteration of treatment at any time will be at the discretion of attending clinicians or the participants themselves. All participants withdrawn from treatment after starting trial treatment will still attend for follow up assessments and complete quality of life (QoL) questionnaires unless unwilling to do so, and Case Record Forms (CRFs) will continue to be completed.

Indications for withdrawal from treatment include:

- - - Clinically significant changes in safety bloods during the study
    - Development of new health problems that will significantly interfere with the study measurements
    - Recurrence of cancer
    - Any AE of Common Terminology Criteria for Adverse Events (CTCAE) grade 3 or higher that is possibly or probably related to trial medication

All participants withdrawn from protocol treatment will still attend for follow-up assessments as per the TRYMS protocol, unless unwilling to do so, and relevant case report forms will continue to be completed and returned to the trial team at the CTRU. If a participant withdraws their consent to all trial procedures (i.e. treatment, follow-up assessments and data collection) their data and samples collected up until that time point will remain on file and will be included in the final study analysis. If a participant withdraws from treatment or any other trial procedures complete a F13 - Withdrawal CRF and return to the CTRU within 7 days.

## Unblinding of participants at the end of treatment

Participants will not be told of their trial allocation until the final analysis has been completed. At this time the CTRU will provide each site with a list of treatment allocations for participants recruited at that site. Participants cannot request their trial allocation until this time.

# ASSESSMENTS / SAMPLES / DATA COLLECTION

Data will be collected using paper CRFs which will be provided by the CTRU and upon completion should be returned to the CTRU at the University of Leeds. Participating hospitals will be expected to maintain a file of essential trial documentation (Investigator Site File), which will be provided by the CTRU, and keep copies of all completed CRFs for the trial in the Investigator Site File.

## Schedule of events

| **Visit (weeks)** | **Written Informed Consent and Registration** | **Eligibility Assessment** | **Baseline (0)** | **Titration (2)** | **Phone 1**  **(6)** | **Interim (13)** | **Phone 2**  **(19)** | **Final (26)** | **Phone 3 (approx.**  **30)c** |
| --- | --- | --- | --- | --- | --- | --- | --- | --- | --- |
| **Bloods** |  | x | xa | xe |  | x |  | xae |  |
| **Local testosterone measurement** |  | x |  |  |  |  |  |  |  |
| **Local FBC, PSA** |  | x |  |  |  | x |  | x |  |
| **Local U+E, LFT, Calcium** |  | x |  |  |  |  |  |  |  |
| **Medical history** |  | x |  |  |  |  |  |  |  |
| **Randomisation** |  |  | x |  |  |  |  |  |  |
| **Concomitant medications** |  | x | x |  | x | x | x | x |  |
| **Blood pressure** |  | x |  |  |  |  |  |  |  |
| **Local lipids, glucose, insulin** |  |  | x |  |  |  |  | x |  |
| **QoL questionnaires** |  |  | x |  |  | x |  | x |  |
| **Height and weight**  **To calculate Body Mass Index (BMI)** |  | x | x |  |  | x |  | x |  |
| **Waist circumferenceb** |  |  | x |  |  | x |  | x |  |
| **Whole body DXA** |  |  | x |  |  |  |  | x |  |
| **AE collection** |  | xd | xd |  | x | x | x | x | x |
| **5ml serum sent to Sheffield** |  |  |  | x |  |  |  |  |  |
| **2ml Serum (local storage) for later transport to Sheffield** |  |  | x |  |  |  |  | x |  |
| **Dispensing trial stock** |  |  | x |  |  | x |  |  |  |
| **Returned canisters weighed by pharmacy** |  |  |  |  |  | x |  | x |  |
| **Phone contact** |  |  |  |  | x |  | x |  | x |

a fasting samples taken in the morning between 8am and 10am. The participant should not have eaten since midnight.

b measured at midpoint between costal margin and iliac crest three times and mean recorded

c telephone contact should be made 30 days after the end of treatment to ensure that all Adverse Events are recorded

d SAEs should be reported from the time of registration and AEs will be collected from the time of randomisation via the CRFs

***etaken between 8 am and 10 am (+/- 1 hour) and 2 hours after application of gel (up to 4 hours after application of gel will be allowed). The phlebotomy area should be washed with soap and water prior to the blood sample being taken.***

TRYMS_V6.8_14_06Jul_04_SponsorID STH15216

Page 28 of 52

## Assessment of eligibility, registration and randomisation

Participants should be screened and have given written informed consent before they are registered and any trial specific procedures performed.

After informed consent and registration the participants’ eligibility should be formally assessed and confirmed:

- Medical history, including concomitant medications, will be taken to confirm eligibility
- Height in centimetres and weight in kilograms (in light indoor clothing and without shoes) will be measured to ensure eligibility on BMI criteria
- Blood pressure (up to 3 measurements recorded after resting. Repeat measurement is not necessary if the first value is below 160/100)
- Blood will be taken to assess eligibility by measurement of serum testosterone (between 8 am and 10 am (+/- 1 hour)), and to assess safety for randomisation (FBC, U+E, LFT, calcium, PSA).

It is vital that the blood sample confirming eligibility is taken within the stated timeframe above due to the natural fluctuations in testosterone during the day. Samples taken before 7 am or after 11am will not be accepted at randomisation. Samples with missing information on the time the sample was taken will also not be accepted at randomisation.

Participants should be randomised no later than 35 days after the date that the eligibility blood sample was taken.

If, following randomisation, a participant is found to be in breach of the eligibility criteria the F16 - Protocol Violations CRF should be completed and faxed to the CTRU immediately.

## Treatment Assessments

All Treatment Assessments need to be booked for morning clinics as bloods must be taken between 8am and 10am (+/- 1 hour). Blood samples at baseline and 26 weeks should be fasting samples with the participant fasting since midnight.

### Assessments required at baseline

The baseline assessments should be performed **after confirmation of eligibility and full trial consent** and **no longer than 35 days before randomisation** and the start of trial treatment. Wherever possible it is recommended that all baseline tests are arranged for one day and the participant should be randomised the morning of the visit so pharmacy can prepare the trial supply for dispensing on the same day.

- - - - Quality of Life: SF36-v2™†, Rosenberg’s Self-Esteem (RSE), Functional Assessment of Chronic Illness Therapy (FACIT) fatigue scale and Derogatis Interview for Sexual Functioning – II Self-Report-Male (DISF-SR II) questionnaires
      - Waist circumference measured in cm measured at midpoint between costal margin and iliac crest three times and mean recorded
      - Height in centimetres and weight in kilograms (in light indoor clothing and without shoes) for BMI calculation
      - Whole body dual energy X-ray absorptiometry (DXA) to assess truncal fat mass, lean body mass, whole body fat mass and bone density
      - Fasted blood sampling between 8 am and 10 am (+/- 1 hour) for :
        - local measurement of lipid profile (total cholesterol (TC), high-density lipoprotein (HDL), low-density lipoprotein (LDL), triglycerides (TG)), insulin and glucose (not time dependent)
        - 2ml serum samples should be taken between 8 am and 10 am (+/- 1 hour) and stored locally for later central measurement of testosterone, oestradiol, Sex Hormone-Binding Globulin (SHBG), Luteinizing Hormone (LH) and Follicule-Stimulating Hormone (FSH) and central storage. These samples should be spun, stored in a 1 x 1ml and 2 x 0.5ml aliquots as per the laboratory handbook and kept at -80°C until the end of the trial.
      - Concomitant medications in accordance with the CRFs
      - It is recommended that the first box of 6 canisters of trial treatment are dispensed at the baseline visit
      - Treatment should be started within 2 days of randomisation

†SF-36v2™ Health Survey © by QualityMetric Incorporated – all rights reserved. SF-36v2 is a trademark of Quality Metric Incorporated.

### Assessments required after 2 weeks of treatment (+/- 2 days)

- - - - A 5ml blood sample should be taken.
      - This sample is non-fasting
      - The sample must be taken between 8 am and 10 am (+/- 1 hour) and 2 hours after application of the gel (up to 4 hours after application of gel will be allowed)
      - Samples taken after less than 12 days of gel application or outside of the time windows specified will not be accepted.
      - This should be sent to the central laboratory at the Royal Hallamshire Hospital for central analysis. The sample should be packaged and posted according to the study laboratory manual and in accordance with local laboratory processes.
      - Dosing confirmation will be returned to the local study team by fax, and should be reviewed by a medically qualified person to ensure that the suggested dose adjustment is clinically appropriate. Following this confirmation the dosing confirmation should be communicated to the participant by telephone *and* in writing using the template letter found in the Investigator Site File.
      - It is possible that a repeat 5ml blood sample may be required within four weeks of the first titration test.
      - The independent endocrinologist may request additional measurement of oestradiol on titration or retest samples to determine if out of range testosterone results are likely to be the result of sample contamination.

### Telephone contact after 6 (± 1) weeks of randomisation

- - - - Participants will be telephoned by a member of the local study team to check on continuing participation
      - Adverse event reporting including pregnancies of partners
      - Concomitant medications in accordance with the CRFs

### Assessments required after 13 (± 1) weeks of randomisation

- - - - Quality of Life: SF36™, RSE, FACIT fatigue and DISF-SR II questionnaires
      - Height in centimetres and weight in kilograms (in light indoor clothing and without shoes) for BMI calculation
      - Waist circumference measured in cm measured at midpoint between costal margin and iliac crest three times and mean recorded
      - Blood sampling (not fasted or timed) for local measurement of full blood count and PSA
      - Adverse event reporting including pregnancies of partners
      - Concomitant medications in accordance with the CRFs
      - 1 box of 6 canisters of Tostran /placebo dispensed (kit code given via the CTRU 24- hour telephone service)
      - Used treatment canisters to be returned to pharmacy and weighed to record treatment compliance

### Telephone contact after 19 (± 1) weeks of randomisation

- - - - Participants will be telephoned by a member of the local study team to check on continuing participation
      - Adverse event reporting including pregnancies of partners
      - Concomitant medications in accordance with the CRFs

### Assessments required after 26 (± 1) weeks of randomisation

- - - - Quality of Life: SF36™, RSE, FACIT fatigue and DISF-SR II questionnaires
      - Participant Blinding Questionnaire: participant self-reported questionnaire to ask the participant’s opinion of their trial treatment at the end of the treatment period
      - If the participant is unable to attend for the 26 week clinic visit within 27 weeks post randomisation, the Quality of Life questionnaires and the Participant Blinding Questionnaire should be provided to the participant by post as soon as possible after the 26 week post randomisation date.
      - Research Team Blinding Questionnaire: questionnaire to be completed by a team member involved in the participant’s clinical care at the end of the treatment period and once all CRFs for the patient have been completed
      - Fasted blood sampling between 8 am and 10 am (+/- 1 hour) for :
        - local measurement of FBC and PSA, lipid profile (TC, HDL, LDL, TG), insulin and glucose (not time dependent)
        - 2ml serum samples should be taken between 8 am and 10 am (+/- 1 hour), 2 hours after application of the gel (up to 4 hours after application of gel will be accepted) and stored locally for later central measurement of testosterone, oestradiol, SHBG, LH and FSH and central storage. These samples should be spun, stored in 1 x 1ml and 2 x 0.5ml aliquots as per the laboratory handbook and kept at -80°C until the end of the trial.
      - Adverse event reporting including pregnancies of partners
      - Whole body dual energy X-ray absorptiometry (DXA) to assess truncal fat mass, lean body mass, whole body fat mass and bone density
      - Height in centimetres and weight in kilograms (in light indoor clothing and without shoes) for BMI calculation
      - Waist circumference measured in cm measured at midpoint between costal margin and iliac crest three times and mean recorded
      - Concomitant medications in accordance with the CRFs
      - Used treatment canisters to be returned to pharmacy and weighed to record treatment compliance

### Telephone contact 30 days after finishing treatment

- - - - Participants will be telephoned by a member of the local study team to check if the participant has experienced any AEs, in accordance with section 12, in the 30 days after finishing treatment

## Sample Handling

In order to maintain the blinding of the trial serum testosterone levels should not be measured locally after the sample that is used to confirm eligibility.

A 5ml blood sample should be sent to the at the Royal Hallamshire Hospital 2 weeks after the participant starts treatment. The sample should be packaged and posted according to the study laboratory manual and in accordance with local laboratory processes.

2ml of serum should be obtained at the baseline and 26 week visits and stored at local sites. These samples should be spun, stored in 1 x 1ml and 2 x 0.5ml aliquots in a secure location and kept at -80°C until the samples are requested to be sent which may be up until the end of the trial. At the end of the trial all samples not previously requested will be sent to the Royal Hallamshire Hospital. These samples should be packaged and posted according to local laboratory processes. The 1ml aliquot from each time point will be analysed to measure testosterone, oestradiol, SHBG, LH and FSH levels and the other two 0.5ml serum aliquots will be stored in the HTA licensed Sheffield Biorepository. These samples will only be used for purposes related to the main trial including later measurement of additional biomarkers and hormones which the investigators decide to be necessary for a more complete explanation of the study results (e.g. adipokines, other androgens). The samples will be destroyed after the final trial publication.

All samples will be identified by trial number, participant initials and date of birth.

## Adverse and Serious Adverse Events

Adverse events (AEs) will be reported at 6, 13, 19 and 26 weeks after randomisation. A further follow up telephone call should also be made to the participant 30 days after they finish trial treatment to ensure that AEs of specific interest and/or severity are recorded in accordance with section 12 of the protocol. Information about AEs, whether volunteered by the participant, discovered by investigator questioning or detected through physical examination, laboratory test or other investigation should be collected and recorded on the CRF.

Information regarding the reporting of Serious Adverse Events (SAEs), Suspected Serious Adverse Reactions (SSARs) and Suspected Unexpected Serious Adverse Reactions (SUSARs) can be found in section 12.4.

## Reporting of pregnancies

Pregnancies of partners of trial participants occurring after randomisation and within the trial period (26 weeks (±1 week) of treatment + 30 days safety follow-up period) should be reported using the Pregnancy Reporting CRF and reported to the CTRU within 24 hours of becoming aware of the pregnancy. Any pregnancies reported during the trial period will be followed up until the outcome is known.

## Deaths

All deaths occurring after randomisation and within the trial period (26 weeks (±1 week) of treatment + 30 days safety follow-up period) must be recorded on the Notification of Death CRF and reported to the CTRU within 24 hours of becoming aware of the death. The date of death and cause of death will be collected.

If the death fulfils the criteria of a Serious Adverse Event (SAE) then an SAE form should also be completed and reported to the CTRU within 24 hours of becoming aware of the event.

## Definition of the end of trial

The end of the trial is defined as the date of the last anticipated data item.

## Central review of DXA scans

Local quality assurance data and a random sample of scan results will be centrally reviewed by a Co-Investigator. These scans will be collected by the CTRU as required.

# PHARMACOVIGILANCE PROCEDURES

## General definitions

### Adverse Events (AEs)

An adverse event (AE) is:

- - - - any unintentional, unfavourable clinical sign or symptom
      - any new illness or disease or the deterioration of existing disease or illness
      - any clinically relevant deterioration in any laboratory assessments or clinical tests.

In addition the following criteria may be used in order to describe *significant adverse events*

which do not meet the criteria for serious (Section 12.1.2):

- - - - requires medical or surgical intervention to prevent permanent impairment of function or permanent damage to body structure.

### Serious Adverse Events (SAEs)

A Serious Adverse Event (SAE) is defined in general as an untoward (unfavourable) event, which is:

- fatal or life-threatening,
- requires or prolongs hospitalisation,
- is significantly or permanently disabling or incapacitating,
- constitutes a congenital anomaly or a birth defect or
- may jeopardise the participant and may require medical or surgical intervention to prevent one of the outcomes listed above.

Events which do not need to be reported as an SAE include hospitalisation for:

- Routine treatment or monitoring of the studied indication not associated with any deterioration in condition.
- Treatment which was elective or pre-planned, for a pre-existing condition not associated with any deterioration in condition, e.g. pre-planned hip replacement operation which does not lead to further complications.
- Any admission to hospital or other institution for general care where there was no deterioration in condition.
- Treatment on an emergency, outpatient basis for an event **not** fulfilling any of the definitions of serious as given above and not resulting in hospital admission.

### Suspected Unexpected Serious Adverse Reactions (SUSARs)

A Suspected Unexpected Serious Adverse Reaction (SUSAR) is a serious adverse drug reaction which also demonstrates the following characteristic of being unexpected:

Unexpected – An adverse event, the nature OR severity of which is NOT consistent with the SmPC.

Events associated with placebo will usually not satisfy the criteria for a SUSAR and therefore expedited reporting. However, where SUSARs are thought to be associated with placebo (e.g. reaction due to excipient or impurity) the Sponsor will report such cases.

## Operational definition and reporting AEs

Adverse events will be collected for all participants and will be evaluated for intensity and causal relationship with the trial medication or other factors according to the National Cancer Institute (NCI) CTCAE V4.0 (NCI-CTCAE). A copy is provided in the Investigator Site File and may be obtained at:

<http://ctep.cancer.gov/protocolDevelopment/electronic_applications/ctc.htm>

Published date: May 28, 2009

AEs of a specific severity defined as CTCAE grade 3 or above, and AEs of particular interest will be collected and recorded on the CRFs. AEs will be collected from randomisation until 30 days after the end of treatment.

Information about AEs, whether volunteered by the participant, discovered by the investigator questioning or detected through physical examination, laboratory test or other investigation will be collected and recorded on the CRF.

### Expected AEs

As this is a blinded trial, adverse events should be assessed for expectedness and causal relationship assuming that the participant has been receiving Tostran.

The following events will be classed as expected AEs within this trial:

The most commonly reported adverse reactions in a controlled clinical study (up to 4 g Tostran) were application site reactions (ASR; 26%) including; paresthesia, xerosis, pruritus and rash or erythema. The majority of these reactions were mild to moderate in severity and diminished or cleared, despite continued application.

| **Organ System** | **Very Common (≥1/10)** | **Common (≥1/100 to <1/10)** |
| --- | --- | --- |
| Blood and lymphatic disorders |  | Increase in haemoglobin and haematocrit |
| Endocrine disorders |  | Increase in male pattern hair distribution |
| Vascular disorders |  | Hypertension |
| Reproductive system and breast disorders |  | Gynaecomastia |
| General disorders and administration site conditions | Administration site reactions | Peripheral oedema |
| Investigations |  | Increased PSA |

### Unexpected AEs

High doses of testosterone treatment are known to commonly reversibly interrupt or reduce spermatogenesis, and reduce testicular size. There are no available data relating to lower dose treatment or Tostran specifically, but we will consider reduced sperm count or reduced testicular size as unexpected AEs.

## Operation definition – Serious Adverse Events

### Expected SAEs

When determining whether an SAE is expected or not, please refer to the SmPC supplied in the Investigator Site File or the latest updated version as instructed by the CTRU. As this is a blinded trial, serious adverse events should be assessed for expectedness and causal relationship assuming that the participant has been receiving Tostran.

## Recording and reporting SAEs, SSARs and SUSARs

All SAEs/SSARs/SUSARs occurring after registration must be recorded on the SAE or SUSAR Form and faxed to the CTRU within 24 hours of the research staff becoming aware of the event. Once all resulting queries have been resolved, the original form should be posted to the CTRU and a copy retained on site. SAEs, SSARs and SUSARs will be collected from registration until 30 days after the end of treatment.

For each SAE / SUSAR the following information will be collected:

- full details in medical terms with a diagnosis, if possible
- event duration (start and end dates if applicable)
- action taken
- outcome
- causality (i.e. relatedness to trial drug / investigation), in the opinion of the investigator
- whether or not the event would be considered expected or unexpected

Any follow-up information should be faxed to the CTRU as soon as it is available. Events will be followed up until the event has resolved, a final outcome has been reached or the trial has reached final analysis.

**Fax Number for reporting SAEs and SUSARs: 0113 343 6774**

## Responsibilities

#### Principal Investigator:

1. Checking for SAEs when participants attend for follow-up visits/treatment.
2. Medical judgment in assigning to SAEs:
   - Seriousness
   - Causality
   - Expectedness (using the SmPC supplied in the Site Investigator File)
3. To ensure all SAEs and SSARs (including SUSARs) are recorded and reported to the CTRU within 24 hours of becoming aware of the event and to provide further follow up information as soon as available. To ensure that all SAEs and SSARS (including SUSARs) are chased with CTRU if a record of receipt is not received within 2 working days of initial reporting.
4. To ensure that all SAEs are recorded and reported to the CTRU in line with the requirements of the protocol.
5. To report SAEs to local committees in line with local arrangements.

#### CTRU:

1. Central data collection and verification of AEs, SAEs, SSARs and SUSARs according to the trial protocol onto a MACRO database.
2. Reporting safety information to the Chief Investigator (CI) or delegate for the ongoing assessment of the risk / benefit according to the Trial Monitoring Plan.
3. Reporting safety information to the independent Data Monitoring and Ethics Committee (DMEC) and Trial Steering Committee (TSC) according to the Trial Monitoring Plan.
4. Expedited reporting of SUSARs, serious breaches of GCP and urgent safety measures to Competent Authority (MHRA in UK), Main Research Ethics Committee (REC) and Sponsor within required timelines.
5. Notifying Investigators of SUSARs that occur within the trial.
6. The unblinding of a participant for the purpose of expedited SUSAR reporting.
7. Preparing Development Update Safety Reports (DSURs) to Competent Authority and Main REC, and blinded safety reports to go to the Sponsor and ProStrakan.
8. Report any SAEs, SSARs, SUSARs and pregnancies to ProStrakan within 2 working

days of receiving initial information

1. Report any urgent safety measures and related protocol amendments to ProStrakan.
2. Respond to queries raised by ProStrakan in relation to any of the above.
3. Checking for (annually) and notifying PIs of updates to the Reference Safety Information for the trial.
4. Preparing standard tables and other relevant information for the DSUR in collaboration with the CI and ensuring timely submission to the MHRA and Main REC.

#### Chief Investigator (or nominated individual in CI’s absence):

1. Clinical oversight of the safety of participants in the trial, including an ongoing review of the risk / benefit.
2. Medical review of all SAEs for seriousness, expectedness and causality and notification to CTRU of the outcome of this review with sufficient time to allow expedited reporting of SUSARs to Competent Authority (MHRA in UK), Main REC and Sponsor within required timelines.
3. Using medical judgement in assigning seriousness, causality and expectedness of SAEs where it has not been possible to obtain local medical assessment.
4. Immediate review of all events assessed as SUSARs in the opinion of the local investigator. In the event of disagreement between local assessment and CI / Sponsor review with regards to SUSAR status, local assessment will not be overruled, but CI / Sponsor may add comments prior to expedited reporting.
5. Review and assign code for all SAEs and SSARs using the Medical Dictionary for Regulatory Activities (MedDRA) Body System Organ Class coding, prior to submission of DSURs.
6. Preparing the clinical sections and final sign-off of DSURs.

#### ProStrakan:

1. Acknowledge receipt of SAE, SSAR, SUSAR and pregnancy data.

#### TSC:

1. In accordance with the Trial Terms of Reference for the TSC, periodically reviewing safety data and liaising with the DMEC regarding safety issues.

#### DMEC:

1. In accordance with the Trial Terms of Reference for the DMEC, periodically reviewing unblinded overall safety data to determine patterns and trends of events, or to identify safety issues which would not be apparent on an individual case basis.

# QUALITY OF LIFE (QOL)

Quality of life will be assessed by participants' self-reported symptoms and functioning using validated instruments completed by participants at appropriate and specified time points throughout the trial. All instruments are self-administered to avoid interviewer bias. The four QoL questionnaires to be used in this study are described below.

The SF-36™, a well validated, multi-purpose, standard health-related QoL evaluation questionnaire, will be used to assess generic QoL. It generates an 8-scale profile of functional

health and well-being scores including a physical functioning scale, as well as summary measures of physical and mental health. This information relates to the previous 4-week time period.

The Functional Assessment of Chronic Illness Therapy Fatigue Scale (FACIT Fatigue Scale) questionnaire has been developed to assess fatigue in participants with chronic illnesses. The FACIT questionnaire has also been validated within participants with any form of cancer as well as other chronic illness conditions as well as long-term survivor samples. The questionnaire relates to the past 7 days and consists of 13 questions relating to the participants’ fatigue.

The Derogatis Interview for Sexual Functioning – II Self-Report-Male (DISF II SR-male version) questionnaire is a 25-item questionnaire that has been designed to provide information about the quality of an individual’s current sexual functioning in quantitative terms. The questionnaire is divided into five domains on sexual drive, sexual arousal, sexual activity, orgasm and partner relationship. The questions refer to the past 30 days. We will also record participants’ current relationship status.

The Rosenberg’s Self-Esteem (RSE) scale has been designed to produce a one-dimensional measure of global self-esteem. Developed in 1965, the questionnaire has been validated and is widely used in social science research. The RSE scale consists of 10 questions which refer to the participants’ general feelings about themselves rather than a specific time period.

## Timing and administration of quality of life questionnaires

Participants will be asked to complete the above described questionnaires (SF36™, FACIT fatigue, DISF-SR II male version and RSE) prior to receiving their first treatment canisters and wherever possible prior to randomisation (baseline), and at 13 and 26 weeks post randomisation. Baseline questionnaires should be given to participants after consent has been obtained and eligibility has been confirmed and must be completed prior to receiving their first treatment canisters and wherever possible prior to randomisation. All questionnaires will be provided in clinic; due to the sensitive nature of these questionnaires participants should be provided with a private area to complete the questionnaires.

Questionnaires should be completed by participants at the time of clinical assessment, but before discussion of the outcome of any medical assessments or blood tests wherever possible. Participants will be asked to seal the questionnaires in pre-supplied envelopes prior to being given to research staff. Research staff will then send the sealed envelopes to the CTRU for entry into the database.

# ENDPOINTS

## Primary endpoints

The primary endpoints are truncal fat mass and the SF36™ physical functioning scale at 26 weeks. Truncal fat mass will be measured by whole body dual energy X-ray absorptiometry (DXA). Local quality assurance data and a random sample of scan images will be centrally reviewed by a Co-Investigator to assess the quality of data recorded on the CRF.

## Secondary endpoints

- BMI (at 13 and 26 weeks)
- Fasting insulin: glucose ratio (at 26 weeks)
- Fasting lipids (at 26 weeks)
- LH levels (at 26 weeks)
- Lean body mass and whole body fat mass (at 26 weeks)
- Bone density (at 26 weeks)
- Other QoL scores from the SF36™, FACIT fatigue, DISF-SRII and RSE questionnaires (at 13 and 26 weeks)
- Waist circumference (at 13 and 26 weeks)

Lean body mass, whole body fat mass and bone density will be measured by whole body DXA scans. Fasting lipids, insulin: glucose ratio, and LH levels will be measured by blood samples collected on the day of the treatment assessment visits (LH levels will be centrally measured), whilst BMI will be calculated from participants’ height and weight. Waist circumference will be calculated by measuring the midpoint between costal margin and iliac crest three times in centimetres and recording the mean.

# STATISTICAL CONSIDERATIONS

## Sample size

The sample size calculation is based on the two primary endpoints, truncal fat mass and SF36™ physical functioning scores. The significance levels for the two primary endpoints have not been adjusted to avoid an inflated type I error in this instance, as we consider the co- primary endpoints to be unrelated.

- - 1. **Minimum sample size**

For the SF36™ physical functioning co-primary endpoint, on the basis of clinical consensus, a moderate difference in physical functioning has been deemed to be clinically important. Using the operational definitions by Cohen24 this translates into detection of a standardised effect size of between 0.5 and 0.8. Using 80% power and a 5% (two-sided) significance level, between 52 and 128 participants (26-64 in each treatment arm) are required to detect this degree of superiority using a two-group t-test of equal means using nQuery Advisor® 7.0.

The minimum sample size is based on the co-primary endpoint, truncal fat mass for both serum testosterone groups combined.

For the truncal fat mass co-primary endpoint, on the basis of clinical consensus, a difference of 1.7 kg between the treatment arms in change of truncal fat mass (over 26 weeks) would be deemed to be clinically significant. This would correspond to an overall weight loss of about

4 kg or 5% of total body weight, which is the minimum weight loss estimated to be associated with a reduction in the risk of cardiovascular disease and diabetes (a difference of

2.2 kg was observed between cancer survivors and controls in the preliminary observational study22).

With a common standard deviation of 3 kg for the change in truncal fat mass23 and using 80% power and a 5% (two-sided) significance level, 50 participants are required in each treatment arm to test for this level of superiority using a two-group t-test for the equality of means, using nQuery Advisor® 7.0 bringing the total required sample size to 100. Assuming a 10%

drop out rate, a minimum of 112 participants will be required to be recruited in order to have 100 evaluable participants.

- - 1. **Maximum sample size**

There is no evidence to indicate that the treatment effect will differ depending on baseline testosterone levels however, a trial with a maximum sample size of 268 patients would be powered to ensure sufficient numbers of participants are included in the subgroups defined by serum testosterone (7 – 9.9 nmol/L and 10 – 12 nmol/l) to draw reliable conclusions in the subgroup analysis of frankly hypogondal men and men with borderline low testosterone. In the event of a sample size of less than 268 participants, the treatment effect for each of the subgroups will be estimated in an exploratory manner.

For the SF36™ physical functioning co-primary endpoint, on the basis of clinical consensus, a moderate difference in physical functioning has been deemed to be clinically important. Using the operational definitions by Cohen24 this translates into detection of a standardised effect size of between 0.5 and 0.8. Using 80% power and a 5% (two-sided) significance level, between 52 and 128 participants (26-64 in each treatment arm) are required to detect this degree of superiority using a two-group t-test of equal means using nQuery Advisor® 7.0. Therefore, between 52 and 128 participants are required in each subgroup defined by serum testosterone, bringing the required total sample size to between 104 and 256.

The maximum sample size is based on the co-primary endpoint, truncal mass in each subgroup.

With a common standard deviation of 3 kg for the change in truncal fat mass23 and using 80% power and a 5% (two-sided) significance level, 50 participants are required in each treatment arm to test for this level of superiority using a two-group t-test for the equality of means, using nQuery Advisor® 7.0. 100 participants are therefore required in each subgroup defined by serum testosterone to ensure sufficient numbers are included in the subgroup analysis, bringing the total required sample size to 200.

Based on the observational study by Greenfield et al.25 where 13% and 14% of male cancer survivors had serum testosterone levels of less than 10 nmol/l and 10 to 12 nmol/l respectively, recruitment is expected to be evenly split between the two baseline serum testosterone categories. To account for a possible 10% drop-out rate, questionnaire non- compliance and also a possible imbalance in the recruitment of the two subgroups, for example an approximate 40:60 ratio, 268 participants will be randomised in total.

## Planned recruitment rate

Participants will be identified through standard clinic visits for management of their disease or through disease-specific cancer databases. Suitable sites with DXA access have been identified through liaison with local clinicians. Due to one of the primary endpoints being DXA based, fewer sites will ensure robust quality assurance.

Expected recruitment to TRYMS is based on recruitment in to the preceding observational study by Greenfield et al.25 The entry criteria for TRYMS will capture 27% of the relevant population based on testosterone levels; a consent rate of 50% is expected.

Completing registrations by the end of January 2015, and randomisations at the beginning of March 2015 will ensure that the trial recruits to the minimum target sample size of 112 participants.

Local clinicians were consulted to ensure that this recruitment rate is considered to be realistic and in proportion to audit figures of potentially eligible male participants.

# STATISTICAL ANALYSIS

## General considerations

The statistical analysis of TRYMS is the responsibility of the CTRU Statisticians. The analysis plan outlined in this section will be reviewed and a full statistical analysis plan will be written before any analysis is undertaken and in accordance with current CTRU standard operating procedures.

All analyses will be conducted on the intention to treat (ITT) population, defined as all participants randomised grouped according to the treatment they were randomised to receive. Hypothesis tests will be two-sided and use a 5% significance level. The co-primary endpoints are considered to be unrelated. For this reason, an adjustment of their significance levels to avoid an inflated type I error was not deemed necessary. 26 27

## Frequency of analyses

A DMEC will be set up to independently review data on safety, protocol adherence and recruitment. Interim reports will be presented to the DMEC in strict confidence at, at least, yearly intervals. This committee, in light of the interim data, and of any advice or evidence they wish to request, will advise the Trial Steering Committee if there is proof beyond reasonable doubt that one treatment is better or if there are concerns regarding the safety of the trial. No formal interim analyses are planned hence no statistical testing will take place until final analysis.

Final analysis will take place after all participants have completed 26 weeks of follow-up.

## Endpoint analyses

Differences between treatment arms for truncal fat mass, whole body fat mass, lean body mass and bone density endpoints for both serum testosterone groups combined will be compared using linear regression adjusting for the minimisation factors and relevant baseline measurement.

Differences between the treatment arms for the BMI, waist circumference and quality of life endpoints will be compared using multi-level repeated measures modelling accounting for data at 13 and 26 weeks (assuming missing data at random) adjusting for the minimisation factors and relevant baseline measurement and allowing for time, treatment, treatment-time

interaction (all fixed effects) and for participant and participant-time interaction (random effects).

Differences between the treatment arms for the fasting insulin: glucose ratio and lipid profile endpoints will be compared using linear regression adjusting for the minimisation factors and relevant baseline measurement.

Sensitivity analyses to assess the impact of missing data on the treatment effect will be performed. If missing data patterns suggest data are missing not at random, to allow for differing assumptions about missing data, alternative analyses will also be carried out (e.g. pattern-mixture modelling).

Exploratory subgroup analysis for the serum testosterone baseline value (7-9.9 and 10- 12nmol/l) will also be performed to investigate the treatment effect on outcomes in frankly hypogonadal men and those with low normal testosterone.

To assess whether LH levels predict response to testosterone treatment, exploratory analysis will be performed on the co-primary endpoint of truncal fat mass using linear regression adjusting for the minimisation factors, baseline truncal fat mass, treatment, (baseline / change from baseline to 26 weeks) LH values and the treatment-by-LH interaction. The change in the model deviance will be used to assess the statistical significance of the interaction term.

# DATA MONITORING

## Data Monitoring and Ethics Committee

An independent DMEC will review the safety and ethics of the trial. Detailed unblinded reports, containing information on rates of occurrence of SAEs, SSARs and SUSARS in addition to other safety and recruitment data, will be prepared by the CTRU Statistician for the DMEC at approximately yearly intervals.

## Data Monitoring

Data will be monitored for quality and completeness by the CTRU. Missing data will be chased until it is received, confirmed as not available or the trial is at analysis. The CTRU/Sponsor will reserve the right to intermittently conduct source data verification exercises on a sample of participants, which will be carried out by staff from the CTRU/Sponsor. Source data verification will involve direct access to participant notes at the participating hospital sites and the collection of copies of consent forms and other relevant investigation reports.

## Clinical governance issues

To ensure responsibility and accountability for the overall quality of care received by participants during the trial period, clinical governance issues pertaining to all aspects of routine management will be brought to the attention of the DMEC and TSC and, where applicable, to individual NHS Trusts.

# QUALITY ASSURANCE AND ETHICAL CONSIDERATIONS

## Quality Assurance

The trial will be conducted in accordance with the principles of GCP in clinical trials, as applicable under UK regulations, the NHS Research Governance Framework (and Scottish Executive Health Department Research Governance Framework for Health and Social Care 2006 for studies conducted in Scotland)*,* and through adherence to CTRU Standard Operating Procedures (SOPs)*.*

CTRU and Sponsor have systems in place to ensure that serious breaches of GCP or the trial protocol are picked up and reported. Investigators are required to promptly notify the CTRU of a serious breach (as defined by Regulation 29A of the Medicines for Human Use (Clinical Trials) Regulations 2004 [Statutory Instrument 2004/1031], as amended by Statutory Instrument 2006/1928) that they become aware of. A “serious breach” is a breach which is likely to effect to a significant degree –

1. the safety or physical or mental integrity of the subjects of the trial; or
2. the scientific value of the trial

For further information, the Investigator should contact the Senior Trial Co-ordinator at the CTRU.

## Ethical considerations

The trial will be performed in accordance with the recommendations guiding physicians in biomedical research involving human subjects adopted by the 18th World Medical Assembly, Helsinki, Finland, 1964, amended at the 52nd World Medical Association General Assembly, Edinburgh, Scotland, 2000. Informed written consent will be obtained from the participants prior to registration into the trial. The right of a patient to refuse participation without giving reasons must be respected. The participant must remain free to withdraw at any time from the trial without giving reasons and without prejudicing his further treatment. The trial will be submitted to and approved by a Main REC prior to entering participants into the trial. The CTRU will provide the Main REC with a copy of the final protocol, participant information sheets, consent forms and all other relevant trial documentation.

# CONFIDENTIALITY

All information collected during the course of the trial will be kept strictly confidential. Information will be held securely on paper and electronically at the CTRU. The CTRU will comply with all aspects of the 1998 Data Protection Act and operationally this will include:

- consent from participants to record personal details including name, date of birth, hospital ID, GP name and address
- appropriate storage, restricted access and disposal arrangements for participant personal and clinical details
- consent from participants for access to their medical records by responsible individuals from the research staff or from regulatory authorities, where it is relevant to trial participation
- consent from participants for the data collected for the trial to be used to evaluate safety and develop new research.
- data collection forms that are transferred to or from the CTRU will be coded with a trial number and will include two participant identifiers, usually the participant’s initials and date of birth.
- where central monitoring of source documents by CTRU (or copies of source documents) is required (such as scans or local blood results), the participant’s name must be obliterated by site before sending.
- where anonymisation of documentation is required, sites are responsible for ensuring only the instructed identifiers are present before sending to CTRU.

If a participant withdraws consent from further trial treatment or from further collection of data, their data and samples will remain on file and will be included in the final trial analysis.

The trial staff at the participating sites will be responsible for ensuring that any data / documentation sent to the CTRU is appropriately anonymised as per instructions given by CTRU in accordance with the trial procedures to conform with the 1998 Data Protection Act.

# ARCHIVING

At the end of the trial, data will be securely archived in line with the Sponsor’s procedures for a minimum of 15 years. Data held by the CTRU will be archived in the Sponsor archive facility and site data and documents will be archived at the participating sites. Following authorisation from the Sponsor, arrangements for confidential destruction will then be made.

# STATEMENT OF INDEMNITY

This trial is sponsored by Sheffield Teaching Hospitals NHS Foundation Trust and as such NHS Indemnity arrangements for clinical negligence apply. The NHS has a duty of care to patients treated, whether or not the patient is taking part in a clinical trial, and the NHS remains liable for clinical negligence and other negligent harm to patients under this duty of care.

This is a clinician-led study, involving the use of a licensed drug. There are no arrangements for no-fault compensation; however, usual product liability will be covered by the manufacturer under the Consumer Protection Act 1987.

# STUDY ORGANISATIONAL STRUCTURE

## Responsibilities

**Chief Investigator (CI) –** The CI will have responsibility for the design and set-up of the trial, the investigational drug supply and pharmacovigilance within the trial.

**Clinical Trials Research Unit** (CTRU) – The CTRU will provide set-up and monitoring of trial conduct to CTRU SOPs, the principles of GCP and the GCP Conditions and Principles as detailed in the UK Medicines for Human Use (Clinical Trials) Regulations 2006 including randomisation design and service, database development and provision, protocol development, CRF design, trial design, source data verification, monitoring schedule and statistical analysis for the trial. In addition the CTRU will support main REC, Site Specific Assessment and R&D submissions and clinical set-up, ongoing management including training, monitoring reports and promotion of the trial. The CTRU will be responsible for the

day-to-day running of the trial including trial administration, database administrative functions, data management, safety reporting and all statistical analyses.

**Sheffield Teaching Hospitals NHS Foundation Trust –** Sheffield Teaching Hospitals NHS Foundation Trust will have responsibility for ensuring that appropriate insurance and indemnity arrangements and appropriate contracts and agreements are in place. They are also responsible for central analysis of trial samples in accordance with the trial protocol and distributing IMP to participating sites.

**ProStrakan –** ProStrakan will provide Tostran 2% gel and Tostran gel placebo for all participants in accordance with the trial protocol.

**Royal Free Hospital/University College Hospital London** – Royal Free Hospital in conjunction with University College Hospital London will be responsible for re-labelling trial medication received from ProStrakan and sending this, blinded, to Sheffield Teaching Hospitals NHS Trust.

**Trial Steering Committee (TSC)** – The TSC, with an independent Chair, will provide overall supervision of the trial, in particular trial progress, adherence to protocol, participant safety and consideration of new information. It will include an Independent Chair and no less than two other independent members. The CI and other members of the TMG will attend the TSC meetings and present and report progress. The Committee will meet annually as a minimum.

**Data Monitoring and Ethics Committee (DMEC)** – The DMEC will review the safety and ethics of the trial by reviewing interim data during recruitment and follow-up. The Committee will meet or communicate via teleconference approximately annually.

## Operational Structure

**Chief Investigator (CI)** – The CI is involved in the design, conduct, co-ordination and management of the trial

**Trial Management Group (TMG)** – The TMG, comprising the CI, CTRU team and co- investigators will be assigned responsibility for the clinical set-up, on-going management, promotion of the trial, and for the interpretation of results. Specifically the TMG will be responsible for (i) protocol completion, (ii) CRF development, (iii) obtaining approval from the main REC, (iv) assisting sites with site specific information forms and R&D application,

(v) submitting a CTA application and obtaining approval from the MHRA, (vi) completing cost estimates and project initiation, (vii) appointing and facilitating the TSC and DMEC,

(viii) reporting of serious adverse events, (ix) monitoring of screening, recruitment, treatment and follow-up procedures, (x) auditing consent procedures, data collection, trial end-point validation and database development.

**Clinical Trials Research Unit (CTRU)** – The CTRU will provide set-up and monitoring of trial conduct to CTRU SOPs and the GCP Conditions and Principles as detailed in the UK Medicines for Human Use (Clinical Trials) Regulations including, randomisation design and service, database development and provision, protocol development, CRF design, trial design, source data verification, monitoring schedule and statistical analysis for the trial. In addition the CTRU will support Integrated Research Application System (IRAS) and

National Institute for Health Research (NIHR) Coordinated System for gaining NHS Permission (CSP) submissions and clinical set-up, ongoing management including training, monitoring reports and promotion of the trial. The CTRU will be responsible for the day-to- day running of the trial including trial administration, database administrative functions, data management, safety reporting, maintaining trial blinding and all statistical analyses.

**Trial Steering Committee (TSC)** – The TSC, with an independent Chair, will provide overall supervision of the trial, in particular trial progress, adherence to protocol, participant safety and consideration of new information. It will include an Independent Chair and no less than two other independent members. The CI and other members of the TMG will attend the TSC meetings and present and report progress. The Committee will meet or communicate via teleconference approximately annually.

**Data Monitoring and Ethics Committee (DMEC)** – The DMEC will review the safety and ethics of the trial by reviewing interim data during recruitment and follow-up. The Committee will meet or communicate via teleconference approximately annually.

# PUBLICATION POLICY

The trial will be registered with an authorised registry, according to International Committee of Medical Journal Editors (ICMJE) Guidelines, prior to the start of recruitment.

The success of the trial depends upon the collaboration of all participants. For this reason, credit for the main results will be given to all those who have collaborated in the trial, through authorship and contributorship. Uniform requirements for authorship for manuscripts submitted to medical journals will guide authorship decisions. These state that authorship credit should be based only on substantial contribution to:

- conception and design, or acquisition of data, or analysis and interpretation of data
- drafting the article or revising it critically for important intellectual content
- and final approval of the version to be published
- and that all these conditions must be met ([www.icmje.org](http://www.bmj.com/)).

In light of this, the Chief Investigator, relevant co-investigators and relevant senior CTRU staff who have made a significant contribution to the trial will be named as authors in any publication. In addition, all collaborators will be listed as contributors for the main trial publication, giving details of roles in planning, conducting and reporting the trial.

To maintain the scientific integrity of the trial, data will not be released prior to the end of the trial, either for trial publication or oral presentation purposes, without the permission of the TSC or the CI. In addition, individual collaborators must not publish data concerning their participants, which is directly relevant to the questions posed in the trial until the main results of the trial have been published and following written consent from the Sponsor.

# KEY REFERENCES

1. Maddams J et al. Cancer prevalence in the UK. Thames Cancer Registry and Macmillan Cancer Support. (http://www.thames-cancer- reg.org.uk/news/uk_prevalence_14072008.pdf)
2. Greenfield DM et al. Prevalence and consequences of androgen deficiency in young male cancer survivors in a controlled cross-sectional study. J Clin Endocrinol Metab 2007; 92: 3476-3482
3. Greenfield DM, Walters SJ, Coleman RE et al. Health related QoL, self esteem, fatigue and sex function in young men after cancer: a controlled cross sectional study. Cancer, 2010; 116: 6:1592-601
4. Nakamura T et al. Contribution of visceral fat accumulation to the development of coronary artery disease in non-obese men. Atherosclerosis 1994; 107: 239-246
5. Chan JM et al. Obesity, fat distribution, and weight gain as risk factors for clinical diabetes in men. Diabetes Care 1994;17: 961-969
6. Koster A et al. Waist circumference and mortality Am J Epidemiol 2008; 15: 167:1465- 1475
7. Shalet SM et al. Vulnerability of the human Leydig cell to radiation damage is dependent upon age. J Endocrinol 1988; 120: 161-165
8. Howell SJ et al. Testicular function after cytotoxic chemotherapy: evidence of Leydig cell insufficiency. J Clin Oncol 1999; 17: 1493-1498
9. Whitehead E et al. The effects of Hodgkin’s disease and combination chemotherapy on gonadal function in the adult male. Cancer 1982; 49: 418-422
10. Huddart RA, Norman A. Changes in BMI after treatment of testicular cancer are due to age and hormonal function, and not chemotherapy. Br J Cancer 2003; 89: 1143-1144
11. Howell SJ et al. The impact of mild Leydig cell dysfunction following cytotoxic chemotherapy on bone mineral density (BMD) and body composition. Clin Endocrinol 2000; 52: 609-616
12. Huddart RA et al. Fertility, gonadal and sexual function in survivors of testicular cancer. Br J Cancer 2005; 93: 200-207
13. Marin P et al. Androgen treatment of middle-aged, obese men: effects on metabolism, muscle and adipose tissues. Eur J Med 1992; 1: 329-336
14. Wang C et al. Long-term testosterone gel (AndroGel) treatment maintains beneficial effects on sexual function and mood, lean and fat mass, and bone mineral density in hypogonadal men. J Clin Endocrinol Metab 2004; 89: 2085-2098
15. Snyder J et al. Effects of testosterone replacement in hypogonadal men. J Clin Endocrinol Metab 2000; 85: 2670-2677
16. Wang C et al. Transdermal testosterone gel improves sexual function, mood, muscle strength, and body composition in hypogonadal men. J Clin Endocrinol Metab 2000; 85: 2839-2853
17. Bhasin S et al. Testosterone replacement increases fat-free mass and muscle size in hypogonadal men. J Clin Endocrinol Metab 1997; 82: 407-413
18. Singh AB et al. the effects of varying doses of T on insulin sensitivity, plasma lipids, apolipoproteins and C-reactive protein in healthy young men. J Clin Endocrinol Metab 2002; 87: 136-143
19. Howell, SJ et al. Randomised placebo-controlled trial of testosterone replacement in men with mild Leydig cell insufficiency following cytotoxic chemotherapy. Clin Endocrinol 2001; 55: 315-324
20. Stein KD et al. Physical and psychological long-term and late effects of cancer. Cancer 2008; 112 (Suppl): 2577-2592
21. Howell SJ et al. Fatigue, sexual function and mood following treatment for haematological malignancy: the impact of mild Leydig cell dysfunction. Br J Cancer 2000; 82: 789-793
22. Greenfield DM et al. Prevalence and consequences of androgen deficiencyin young male cancer survivors in a controlled cross-sectional study. J Clin Endocrinol Metab 2007; 92: 3476-3482
23. Snyder J et al. Effects of testosterone replacement in hypogonadal men. J Clin Endocrinol Metab 2000; 85: 2670-2677
24. Cohen J et al. Statistical power analysis for the behavioural sciences. Hillsdale, NJ: Lawrance Earlbaum Asdociates 12-25, 1988
25. Greenfield DM et al. Prevalence and consequences of androgen deficiency in young male cancer survivors in a controlled cross-sectional study. J Clin Endocrinol Metab 2007; 92: 3476-3482
26. Li, QH. Evaluating Co-primary Endpoints Collectively in Clinical Trials. Biometrical Journal 2009. 51: 1: 137–145
27. Turk DC et al. Analyzing multiple endpoints in clinical trials of pain treatments: IMMPACT recommendations 2008. Pain. 139: 485–493

# APPENDIX A - GLOSSARY OF TERMS

AE ASR BP BMI

Adverse Event Annual Safety Report Blood Pressure

Body Mass Index

BSA Body Surface Area

CI CRF CSG CSP

Chief Investigator Case Record Form Clinical Studies Group

Coordinated System for gaining NHS Permissions

CTA Clinical Trials Authorisation

CTCAE Common Toxicity Criteria for Adverse Events CTRU Clinical Trials Research Unit

DISF-SR II DMEC DSUR DXA

E2 eGFR

EudraCT FACIT FBC FSH GCP HDL HTA ICH GCP ICMJE IMP

INR IRAS ISF ITT LDL LFT LH

Derogatis Interview for Sexual Functioning – II Self-Report-Male Data Monitoring and Ethics Committee

Development Safety Update Report Dual-energy X-ray Absorptiometry Estradiol

estimated Glomerular Filtration Rate

European Union Drug Regulating Authorities Clinical Trials Functional Assessment of Chronic Illness Therapy

Full Blood Count

Follicle-Stimulating Hormone Good Clinical Practice

High Density Lipoprotein Human Tissue Authority

International Conference on Harmonisation of Good Clinical Practice International Committee of Medical Journal Editors

Investigational Medicinal Product International Normalised Ratio Integrated Research Application System Investigator Site File

Intention To Treat

Low Density Lipoprotein Liver Function Test Luteinizing hormone

Main REC Main Research Ethics Committee

MedDRA MHRA NCRI NIHR CSP

NHS NIHR PI PIN PSA PSF

Medical Dictionary for Regulatory Authorities Medicine and Healthcare Products Regulatory Agency National Cancer Research Institute

National Institute for Health Research Coordinated System for gaining NHS Permission

National Health Service

National Institute for Health Research Principal Investigator

Personal Identification Number Prostate-Specific Antigen Pharmacy Site File

QoL Quality of Life

RSE SAE SSAR SHBG SOP SmPC SSOP SUSARs TC

TG TMG TSC

tT U+E

Rosenberg’s Self-Esteem Serious Adverse Event

Suspected Serious Adverse Reaction Sex Hormone-Binding Globulin Standard Operating Procedure Summary of Product Characteristics Site Specific Operating Procedure

Suspected Unexpected Serious Adverse Reactions Total Cholesterol

Triglycerides

Trial Monitoring Group Trial Steering Committee Testosterone

Urea and Electrolytes

# APPENDIX B: Clinical Trial Risk Assessment Document

| **Study Title: TRYMS** | | | | | |
| --- | --- | --- | --- | --- | --- |
| **Risks associated with trial interventions 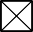** **LOW ≡** Comparable to the risk of standard medical care  **MODERATE ≡** Somewhat higher than the risk of standard medical care  **HIGH ≡** Markedly higher than the risk of standard medical care | | | **Protocol ID: TRYMS** | | |
|  |  |  | **EudraCT No.: 2011-000677-31** | | |
| **Justification:** *Briefly justify the risk category selected and your conclusions below (where the table is completed in detail the detail need not be repeated, however a summary should be given):* This trial has been assigned as a low risk trial as the IMP is being used within the licensed indication and within the dose ranges given within the Summary of Product Characteristics. No modifications are being made to the route of administration and the IMP is being given as a singular agent. The safety profile of Tostran 2% Gel is also well established. | | | | | |
| **What are the key risks related to therapeutic interventions you plan to monitor in this trial?** | | **How will these risks be minimised?** | | | |
| **IMP/Intervention** | **Body system/Hazard** | **Activity** | | **Frequency** | **Comments** |
| Tostran 2% Gel | Increase in Prostate- Specific Antigen (PSA) | Sites will test participants PSA levels at each trial follow-up visit | | Baseline 13 weeks,  26 weeks |  |
| Tostran 2% Gel | Increase in haemoglobin and haematocrit | Sites will measure participants full blood count at each trial follow-up visit | | Baseline, 13 weeks,  26 weeks |  |
|  |  |  | |  |  |
|  |  |  | |  |  |
| **Outline any other processes that have been put in place to mitigate risks to participant safety (e.g. IDMC, independent data review,...)**  A Data Monitoring and Ethics Committee (DMEC) will be convened for the trial who will periodically, on at least an annual basis, review unblinded safety information for the trial. The DMEC will, in light of these reports, have the authority to recommend trial closure to the Trial Steering Committee (TSC) should they have concerns over the safety or ethics of the trial. The TSC have the authority to close the trial at any time.  After 2 weeks of treatment with Tostran 2% Gel or the equivalent placebo gel participants will have their serum testosterone values measured by a central laboratory at the Royal Hallamshire Hospital. As these results could lead to the unblinding of clinical teams at sites the results will be processed by the CTRU and converted in to a dose adjustment before being sent to participating sites. Sites will therefore not be able to see the blood result for their participants. For participants | | | | | |

using Tostran gel the dose adjustment will be based on their actual testosterone levels and will ensure that they are receiving their optimal dose. Participants using the placebo gel will have their dose adjusted at random to ensure the blinding of the trial is maintained. Any testosterone levels that are outside of pre-defined safety limits (<5nmol/l or >40nmol/l) will have their results reviewed by an Independent Consultant Endocrinologist to assess the safety of that participant continuing within the trial.

Participant data will be entered on to a validated database and monitored for completeness and quality by the CTRU. Missing data will be chased until it is received, confirmed as not available, or the trial is at analysis. A validation check program will be incorporated in to the trial database to verify the data, and discrepancy reports will be generated for resolution by the investigator. Priority validations will be incorporated in to the validation programme to ensure that any discrepancies related to participant rights or the safety of participants are expedited to sites for resolution.
