# Supplementary material for: Testosterone replacement in young male cancer survivors: A 6-month double-blind randomised placebo-controlled trial
Source: PLoS Med. 2019 Nov 12;16(11):e1002960. doi: 10.1371/journal.pmed.1002960 (PMC6850530; doi:10.1371/journal.pmed.1002960)
Supplement: Attachment — Submitted filename: Response to Editor and reviewers.Final.docx [file pmed.1002960.s002.docx]

**Response to Editor and reviewers**

**Requests from the editors:**

1. Abstract – Please provide demographic details of participants; the final sentence of the ‘Methods and Findings’ section should include a sentence on the studies limitations; please mention country and setting; please use past tense when you describe the findings

**Response:** We have revised the abstract as suggested on page 2:

- Demographics: “A total of 136 men, 42.6% aged 25 to 37 years and 57.4% 38-50 years, 88% testicular cancer and 10% lymphoma, matched for BMI were recruited and randomised.”
- Study limitations: “The limitations of our study were a relatively short duration of treatment, only three cancer groups included, and no hard endpoint data such as cardiovascular events.”
- Setting: “A multi-centre UK study conducted in specialised oncology hospital setting.”
- We have used past tense and revised throughout manuscript.

2. Funding -I don't think you need to mention the funders in the text

**Response:** This has been removed

3. Please provide an Author Summary – in the main text immediately following the abstract. This should be a non-technical, lay summary. See style for guidelines.

**Response:** We have now provided a structured Author Summary on page 3.

4. Please move the CONSORT flowchart should be in the main paper – usually this would be Table 1 and please reorder others and any shout outs in the text.

**Response:** Consort flowchart is included in the main paper and has been labelled Figure 1, and text updated.

5. Please provide a table of adverse events?

**Response:** Table of adverse events has been added as Table 4.

6. At the start of the discussion, we ask you to generalize less and just say what you found in your study (past tense)

**Response:** We have revised the first paragraph of the discussion as suggested on page 10:

“We demonstrated that six months testosterone treatment in young adult male cancer survivors with borderline low testosterone levels improved body composition. Testosterone treatment was associated with decreased trunk and whole body fat mass of 0.9 and 1.8kg respectively and whole body lean mass increased 1.5Kg. There was no associated change in QoL or metabolic parameters.”

7. why are there two reference lists?

**Response:** This was a mistake and has been corrected.

8. Please provide a CONSORT checklist?

**Response:** Now supplied as supplementary file.

9. Please provide an analysis plan?

**Response:** SAP supplied as supplementary file.

10. Page 10 – you mention multiple questionnaires – are all provided as Supp Files or available without paywall in cited articles? If not, please provide.

**Response:** The questionnaires were all proprietary and referenced in the text.

**Comments from the reviewers:**

**Reviewer #1:** These authors have carried out a detailed analysis of the effects of testosterone replacement in a group of relatively young cancer survivors: the three groups they chose included testicular cancer, lymphoma and leukemia. The study was meticulously organized and performed, in an exemplary randomized double-blind manner. Unfortunately, the outcomes were fairly negative, showing only a minimal change in body fat but few other important questionnaire or metabolic changes. Nevertheless, these are significant novel data in view of the massive increase in cancer survivors and the few large-scale and placebo-controlled studies.

My major comment is that as the results were relatively disappointing in terms of positive effects, it would be helpful if the authors could offer advice as to which patients should in fact be offered treatment. The changes in body fat were very small, but is there is a level of baseline testosterone in which it should be seriously considered? I cannot see in the present manuscript what threshold of testosterone is worthy of active intervention.

**Response:** We accept that the changes in fat mass are relatively small when considering an obese population; however, the population we studied was young male cancer survivors and in this population most are not obese although they have increased fat mass compared to the healthy population and there was no data on the impact of testosterone replacement on body composition. Based on our results we can now state that testosterone replacement in those with borderline low testosterone levels will improve body composition. As suggested by the reviewer we have now changed the final paragraph of the discussion on page 12 to provide a recommendation:

“In conclusion, in young adult male cancer survivors with low and low-normal fasting morning total testosterone less than 12 nmol/l, testosterone treatment is associated with improvement in adverse body composition and the reduction in trunk fat mass with testosterone treatment is potentially more beneficial in those with an increased trunk fat mass. We suggest that in these patients testosterone replacement be considered in the context of other interventions to improve body composition.”

Minor comments;

1. The adherence rates were similar in the two groups, but were rather low; can the authors discuss this further?

**Response:** The adherence is better than reported in most real-world studies. We have now added discussion of adherence in this study on page 11, 2^nd^ paragraph: “We demonstrated better than 60% adherence in more than 60% of participants, assessed by participant reports and weighing of returned canisters. About 4% of participants were shown to have less than 60% adherence. In the remaining participants, we could not confirm adherence due to non-return of canisters. In clinical practice, persistence with topical testosterone treatment is about 35% at 6 months, and the main reason for discontinuation is perceived lack of efficacy [22].”

2. A couple of sentences on the changes in levels of testosterone are repeated on page 8.

**Response:** The repeat has been deleted.

3. The references are given twice, and are not the same.

**Response:** This has been corrected.

4. All units should also be in SI at first mention and thereafter at every point an international audience.

**Response:** All units converted to SI throughout.

**Reviewer #2:** This is a useful RCT on the effect of Testosterone Replacement in Young Male cancer Survivors (TRYMS). The randomisation, sample size calculation, statistical methods and analyses, presentation (tables and figures) and interpretation of the results are mostly adequate and of a good standard. However, there are still a few issue needing attention, especially on the change of the study design and its consequences.

1. I can understand the original plan is to study two strata serum testosterone 200-285 and 286-350 separately to have detailed understanding of the effect of the intervention on these two subgroups. But recruitment difficulty forced the investigators to study a combined group with a broader range of serum testosterone of 200 to 350 instead. The samples size calculation for this combined group is still fine. some issues here: a) where was the effect size of 1.7kg from? references? Were effect sizes same for both the 200-285 and 286-350 groups? b) A sample size of 100 (112 including 10% dropouts) of SF36-PF are only good for effect size 0.6 to 0.8 not the moderate 0.5, so basically only good for detecting relatively bigger effect. c) The final sample size of 136 makes it slightly better but feels like this is a RCT failing to recruit enough patients so failed original purposes and then used the combined group as a compromise. However, the pros and cons of the change in study design and its consequences need to be clearly discussed in the discussion.

**Response:** The difference of 1.7 kg was based on clinical consensus. This difference would correspond to an overall weight loss of about 4 kg or 5% of total body weight, which is the minimum weight loss estimated to be associated with a reduction in the risk of cardiovascular disease and diabetes (a difference of 2.2 kg was observed between cancer survivors and controls in the preliminary observational study). We have added clarification that the difference was based on clinical consensus and that there was no evidence to indicate that the treatment effect would differ depending on baseline serum testosterone levels. The sample size required to detect a moderate effect size for SF36-PF ranged from 48 to 128 per serum testosterone strata. Due to the uncertainty in the anticipated effect size the decision was made to base the sample size on truncal fat mass in the original design and this decision was carried forward at the redesign stage. As the reviewer points out, the decision to continue recruitment beyond the minimum sample size allowed for the potential to reach the sample size of 144, accounting for 10% drop out. We have added this to the sample size calculation.

**The following has been added to the statistical methods (Page 7 paragraph 3):**

“The sample size calculations were based on a two-sample t-test. The two primary endpoints were deemed unrelated, so alpha was not split between the endpoints. Although there was no evidence to indicate that the treatment effect would differ depending on baseline serum testosterone levels, the trial was originally powered to recruit and analyse in two strata defined by eligibility serum testosterone 7.0 to 9.9 and 10.0 to 12.0 nmol/l to test for a treatment effect in each stratum. For trunk fat mass, an effect size of 1.7 kg was determined to be clinically meaningful based on clinical consensus. With a common standard deviation of 3 kg for the change in trunk fat mass [17], 80% power and a 5% (two-sided) significance level, 50 participants were required per group (total 200 participants). For the SF36-PF, a moderate difference (standardised effect size of 0.5 to 0.8) was deemed to be clinically meaningful. Between 52 and 128 participants per stratum were required to detect this degree of superiority using a two-group t-test of equal means with 80% power and a 5% (two-sided) significance level (total 104 to 256 participants). Due to the large range in sample size requirements for SF36-PF, the decision was made to base the final sample size on trunk fat mass. Therefore, after accounting for a 10% drop-out rate and a possible imbalance of up to 40:60 in the two strata, the required sample size was 268. Recruitment to TRYMS was slower than predicted, so a design modification was made to combine the two strata, and investigate the influence of baseline serum testosterone in an exploratory analysis. The decision to modify was made without reference to any outcome data. To detect a 1.7 kg difference in trunk fat mass, assuming a dropout rate of 10%, the revised minimum sample size was determined to be 112. A corresponding sample size of between 58 and 144 was required to detect a moderate effect size of 0.5 to 0.8 in SF36-PF assuming a drop out of 10%.”

Whilst the decision to combine the two groups was in light of poor recruitment, the original decision to have a focussed approach to recruiting patients in the two groups was borne out of concern that the trial would only recruit participants with borderline low testosterone. At the time of redesign, the number of participants recruited in each of the serum testosterone strata was found to be reasonably balanced and the pragmatic decision was made to redesign the trial.

**The following has been added to the discussion (page 12 first paragraph):**

“The study did not recruit to the original sample size; however, on the basis that there was no clinical reason to suspect a difference in treatment effects between groups, a redesign to combine serum testosterone groups was made which led to a minimum target of 112 participants. The trial also recruited 136 participants which was close to the maximum number of participants required for the SF36-PF endpoint on the continuous scale. Although the study was adequately powered for the primary endpoint of trunk fat mass after the redesign, and minimisation factors were used, there is a possibility in a trial of this size there was some imbalance between groups which could have influenced the outcome.”

2. Sample size and analysis of SF36-PF as co-primary endpoint are not adequate. Firstly the sample size is slightly underpowered for SF36-PF; secondly, to change from a continuous outcome (powered) to binary outcome (not powered and a bit odd using 100 vs <100) makes the analysis not powered and not planned. Therefore the results for SF36-PF are inconclusive and need to tone down the claims of the non-significant findings due to all these compromises.

**Response**: We have now added the following to the discussion of the QoL results (page 11, paragraph 3): “We found no effect of testosterone treatment on quality of life assessed with several questionnaires, although because the SF-36 data were heavily skewed, which required a change to the statistical analysis plan for this outcome, we cannot definitively confirm that there was no effect on this score.”

3. One minor point on table 1, there are many missing % signs for the values in the table.

**Response**: We have added the missing % signs. Thank you.
